# Characterization of Metal Tolerance Proteins and Functional Analysis of GmMTP8.1 Involved in Manganese Tolerance in Soybean

**DOI:** 10.3389/fpls.2021.683813

**Published:** 2021-11-29

**Authors:** Jifu Li, Rongshu Dong, Yidan Jia, Jie Huang, Xiaoyan Zou, Na An, Jianling Song, Zhijian Chen

**Affiliations:** ^1^Institute of Tropical Crop Genetic Resources, Chinese Academy of Tropical Agricultural Sciences, Haikou, China; ^2^College of Tropical Crops, Hainan University, Haikou, China

**Keywords:** metal tolerance protein, cation diffusion facilitator, Mn transporter, Mn toxicity, Mn detoxification, *Glycine max*

## Abstract

Manganese is an essential micronutrient for plant growth but can be toxic to plants when it reaches excessive levels. Although metal tolerance proteins (MTPs), which belong to the cation diffusion facilitator (CDF) family, have been demonstrated to play critical roles in manganese (Mn) tolerance in plants, the characteristics and functions of GmMTP members in the response of soybean (*Glycine max*) to Mn toxicity have not been documented. In this study, growth inhibition was observed in soybean plants that were exposed to a toxic level of Mn in hydroponics, as reflected by the generation of brown spots, and decreased leaf chlorophyll concentration and plant fresh weight. Subsequent genome-wide analysis resulted in the identification of a total of 14 *GmMTP* genes in the soybean genome. Among these *GmMTPs*, 9 and 12 were found to be regulated by excess Mn in leaves and roots, respectively. Furthermore, the function of *GmMTP8.1*, a Mn-CDF homologue of *ShMTP8* identified in the legume *Stylosanthes hamata* that is involved in Mn detoxification, was characterized. Subcellular localization analysis showed that GmMTP8.1 was localized to the endoplasmic reticulum (ER). Heterologous expression of *GmMTP8.1* led to the restoration of growth of the Mn-hypersensitive yeast (*Saccharomyces cerevisiae*) mutant Δ*pmr1*, which is made defective in Mn transport into the Golgi apparatus by P-type Ca/Mn-ATPase. Furthermore, *GmMTP8.1* overexpression conferred tolerance to the toxic level of Mn in Arabidopsis (*Arabidopsis thaliana*). Under excess Mn conditions, concentrations of Mn in shoots but not roots were decreased in transgenic Arabidopsis, overexpressing *GmMTP8.1* compared to the wild type. The overexpression of *GmMTP8.1* also led to the upregulation of several transporter genes responsible for Mn efflux and sequestration in Arabidopsis, such as *AtMTP8*/*11*. Taken together, these results suggest that GmMTP8.1 is an ER-localized Mn transporter contributing to confer Mn tolerance by stimulating the export of Mn out of leaf cells and increasing the sequestration of Mn into intracellular compartments.

## Introduction

As one of the essential micronutrients for plant growth, manganese (Mn) acts as an activator of many enzymes, serving various functions in a set of physiological and biochemical processes (Millaleo et al., 2010; Marschner, 2012; Long et al., 2021). Being a trace element, Mn is required by plants at a low dose and can cause phytotoxicity when present in excess (Millaleo et al., 2010; Shao et al., 2017). Mn toxicity is an important factor limiting plant growth on acid, poorly drained soils, and sterilized soils. Previous studies have shown that excess Mn can trigger oxidative stress by the generation of reactive oxygen species (ROS) (e.g., OH and O_2_^–^) from Mn ions *via* the Fenton reaction, resulting in disrupted metabolic pathways and damaged thylakoid membrane structure and macromolecules in plant cells (Millaleo et al., 2010; Dragišić Maksimović et al., 2012). Therefore, Mn toxicity inhibits enzyme activity, declines chlorophyll biosynthesis and photosynthesis, and impairs the uptake and translocation of other mineral elements (Fernando and Lynch, 2015; Li et al., 2019). In general, visible symptoms of Mn toxicity for most plants include generation of brown spots, chlorosis, and necrosis in leaves, ultimately inhibiting plant growth (Millaleo et al., 2010; Li et al., 2019). Thus, better understanding of mechanisms underlying the response of plants to Mn toxicity is of great importance for breeding Mn-tolerant crop varieties.

To cope with Mn toxicity, plants have developed comprehensive mechanisms to regulate Mn homeostasis by mediating Mn uptake, efflux, and trafficking at both cellular and tissue levels (Shao et al., 2017; Alejandro et al., 2020). For example, the cation diffusion facilitator (CDF) family is an integral membrane divalent cation transporter that plays critical roles in metal tolerance by regulating metal homeostasis in plant cells (Migocka et al., 2014, 2015). Most CDF proteins include an N-terminal signature sequence, a conserved C-terminal cation efflux domain, and several transmembrane domains (TMDs), which are critical for metal transport activities (Montanini et al., 2007; Chen X. et al., 2016). CDF transporters are involved in the transport of divalent cations, such as zinc (Zn), iron (Fe), and Mn, from the cytoplasm to extracellular space or to subcellular compartments (Gustin et al., 2011). Thus, the CDF members identified from numerous organisms can be classified into three major substrate-specific clusters, namely, Zn-CDF, Fe/Zn-CDF, and Mn-CDF, according to their transported substrate specificities (Montanini et al., 2007). Plant CDF proteins are generally designated as metal tolerance proteins (MTPs), which can be further categorized into seven subgroups based on the initial phylogenetic analysis and annotation of the Arabidopsis (*Arabidopsis thaliana*) MTP family (Gustin et al., 2011). For example, of the 12 MTP members in Arabidopsis, six AtMTPs (AtMTP1, AtMTP2, AtMTP3, AtMTP4, AtMTP5, and AtMTP12) are assigned to groups 1, 5, and 12 within the Zn-CDF cluster, while AtMTP6 and AtMTP7 belong to groups 6 and 7 within the Fe/Zn-CDF cluster. The remaining four AtMTPs (AtMTP8, AtMTP9, AtMTP10, and AtMTP11) are categorized into groups 8 and 9 within the Mn-CDF cluster (Gustin et al., 2011).

Among the three substrate-specific CDF clusters, MTP members of groups 8 and 9 within the Mn-CDF cluster have been characterized to be involved in regulating Mn tolerance either by sequestration of Mn or export of Mn out of the cell. For example, group 8 includes ShMTP8 from *Stylosanthes hamata*, AtMTP8 from Arabidopsis, OsMTP8.1/8.2 from rice (*Oryza sativa*), and CsMTP8 from cucumber (*Cucumis sativus*). These MTP members exhibit substrate-specificity to Mn and are implicated in the transport of Mn into vacuoles (Delhaize et al., 2003; Chen et al., 2013; Migocka et al., 2014; Eroglu et al., 2016; Takemoto et al., 2017). In addition, a variety of MTP members of group 9 have also been involved in the regulation of Mn homeostasis, as observed in CsMTP9 from cucumber, OsMTP9/11 from rice, BmMTP10/11 from *Beta vulgaris* ssp. *maritima*, AtMTP11 from Arabidopsis, and PtMTP11.1/11.2 from poplar (*Populus trichocarpa*); these MTPs are localized to the plasma membrane, Golgi apparatus, or pre-vacuolar compartments and have also been documented to regulate Mn homeostasis and tolerance (Delhaize et al., 2007; Peiter et al., 2007; Erbasol et al., 2013; Migocka et al., 2015; Ueno et al., 2015; Tsunemitsu et al., 2018b). Although the conserved functions of MTPs within the Mn-CDF cluster indicate similar roles of MTPs in Mn detoxification, it is still unknown whether MTP homologues in this specific cluster from different plants fulfill the same function and contribute to Mn tolerance.

Soybean (*Glycine max*) is recognized as one of the economically important nitrogen fixing legume crops and provides more than 60% of high-quality protein and oil for humans and animals in the world (Herridge et al., 2008; Lu et al., 2017). Although several studies have been conducted to investigate the response of soybean to Mn toxicity (Lavres et al., 2009; Yang et al., 2009; Chen Z. et al., 2016; Santos et al., 2017; Liu et al., 2020), underlying molecular mechanisms, through the involvement of MTPs, have not been elucidated. Key participants within the specific Mn-CDF cluster involved in Mn detoxification in soybean remain undetermined. In this study, a total of 14 MTP members were identified in the soybean genome. The expression pattern of each *GmMTP* gene in response to various metal treatments was analyzed. Furthermore, GmMTP8.1 was identified as a novel MTP8-like protein and displayed high similarity with ShMTP8 from *S. hamata* within the Mn-CDF cluster. Thus, GmMTP8.1 was studied in more detail to characterize its potential functions in Mn detoxification.

## Materials and Methods

### Plant Materials and Growth Conditions

The soybean cultivar Zhonghuang 13 was used in this study. Seeds were surface-sterilized with 10% (v/v) H_2_O_2_ and germinated in paper rolls moistened with a half-strength Hoagland nutrient solution, and were cultivated in a growth chamber under conditions of 14-h light at 26°C/10-h dark at 23°C, 120 μmol m^–2^ s^–1^ photon flux density, and 70% relative humidity. After seed germination for 7 days, the soybean seedlings were transferred into a 15-L hydroponic box containing a 13-L full-strength Hoagland nutrient solution, as described by Chen Z. et al. (2016). The Hoagland nutrient solution contained 1500 μM KNO_3_, 1200 μM Ca(NO_3_)_2_, 400 μM NH_4_NO_3_, 500 μM KH_2_PO_4_, 1000 μM MgSO_4_, 25 μM MgCl_2_, 300 μM K_2_SO_4_, 300 μM (NH_4_)_2_SO_4_, 5 μM MnSO_4_, 0.38 μM ZnSO_4_, 1.57 μM CuSO_4_, 0.09 μM (NH_4_)_6_Mo_7_O_24_, 2.5 μM NaB_4_O_7_, and 80 μM Fe-EDTA. The pH value of the solution was adjusted to 5.8 using HCl or KOH every 2 days. The solution was aerated every 20 min per hour and refreshed weekly. Plants were cultivated in a greenhouse at temperatures ranging from 2 to 30°C under natural sunlight with a photoperiod of about 14 h and 50-70% relative humidity. Each hydroponic box containing four soybean seedlings served as one biological replicate, and the experiment included three biological replicates. After 14 days of growth, leaves, stems, and roots were separately cut from the plants. Samples from two plants of each biological replicate were pooled for gene expression analysis. All the samples were frozen with liquid nitrogen, and then stored at −80°C prior to RNA extraction.

To investigate the effects of excess Mn on soybean growth, after seed germination for 7 days, the seedlings were transferred to the full-strength Hoagland nutrient solution for 7 days as described above. Then, 14-day-old seedlings were transferred into the nutrient solution supplied with 5, 50, or 100 μM MnSO_4_, according to Chen Z. et al. (2016). Five μM MnSO_4_ was used as the control in this experiment. Each hydroponic box containing four soybean seedlings served as one biological replicate, and each treatment included three biological replicates. After 7 days of Mn treatments, shoots or roots from two plants of each biological replicate were pooled for determination of plant fresh weight and Mn concentration. A soil plant analysis development (SPAD)-502 chlorophyll meter (Konica-Minolta, Japan) was used to detect chlorophyll concentration according to the instructions of the manufacturer. Leaves or roots from the remaining two plants of each biological replicate were pooled for gene expression analysis. The samples were then stored at −80°C prior to RNA extraction.

To analyze the effects of excess Fe, Zn, and copper (Cu) stress on gene expression, 14-day-old seedlings pre-cultured in the nutrient solution as described above were transferred into a fresh nutrient solution containing 800 μM Fe-EDTA, 20 μM ZnSO_4_, or 20 μM CuSO_4_, which were regarded as excess Fe, Zn, and Cu stress, respectively. Soybean seedlings grown in the full-strength nutrient solution were set as the control. Each hydroponic box containing four soybean seedlings served as one biological replicate, and the experiment included three biological replicates. After 7 days of metal treatments, samples of leaves or roots were separated from two plants of each biological replicate, and were then pooled for gene expression analysis. The samples were frozen with liquid nitrogen and stored at −80°C prior to RNA extraction.

### Identification of Soybean *GmMTP* Genes

The sequences of 12 *MTP* genes in Arabidopsis obtained from the NCBI website^1^ were used separately as queries for BLAST search against the soybean genome in the Phytozome website.^2^ After removing redundant sequences, a total of 14 *GmMTP* members were identified in the soybean genome that contained the cation efflux domain (PF01545). Subsequently, these candidates were named from *GmMTP4.1* to *GmMTP11.2* based on the phylogenetic relationship and sequence identity between GmMTP and Arabidopsis AtMTPs. General information for each *GmMTP* gene was obtained from the Phytozome website. The protein molecular weight of each GmMTP member was calculated using the ExPASy software.^3^ Gene Structure Display Server^4^ was used for gene structure analysis. The Pfam and MEME programs were used to analyze GmMTP conserved domains and motifs, respectively, according to Liu et al. (2018). TMDs were predicted by the TMHMM Server v.2.0. Construction of the phylogenetic tree was based on entire protein sequence alignments through ClustalX using the method neighbor-joining with 1,000 bootstrap replicates with MEGA 4.1.^5^ The sequences of MTPs from other plant species were retrieved from corresponding databases, as described previously (Gustin et al., 2011; Migocka et al., 2014; Li et al., 2018; Liu J. et al., 2019).

### Quantitative Real-Time Polymerase Chain Reaction Analysis of *GmMTP* Genes

Total RNA was extracted using TRIzol Reagent (Invitrogen, United States) according to the instructions of the manufacturer. First-strand cDNA was synthesized using a HiScript III cDNA (Vazyme, China) synthesis kit. SYBR Green Master Mix (Vazyme, China) and QuantStudio™ 6 Flex Real-Time System (Thermo Fisher Scientific, United States) were used for quantitative real-time polymerase chain reaction (qRT-PCR) analysis. qRT-PCR reaction was performed as follows: 95°C for 1 min, 40 cycles of 95°C for 15 s, 60°C for 15 s, and 72°C for 30 s. Fluorescence data were collected at 72°C. The qRT-PCR primers of genes are summarized in Supplementary Table 1. Relative gene expressions were calculated relative to the expression levels of the housekeeping gene, *GmEF-1a* or *AtEF-1a*. Gene expression analysis included three biological replicates.

### Subcellular Localization of GmMTP8.1

The open reading frame (ORF) of *GmMTP8.1* without stop codon was amplified by PCR from root cDNA using *GmMTP8.1-GFP-F/R* primers (Supplementary Table 1). The amplified product was digested with *Eco*RI and *Bam*HI, and was then subcloned into the same sites after the cauliflower mosaic virus (CaMV) 35S promoter of the binary vector *pBWA(V)HS-GLosgfp* and fused with the N-terminal of green fluorescent protein (GFP). The *GmMTP8.1-GFP* construct and empty vector were introduced separately into *Agrobacterium tumefaciens* strain Gv3101 using the freeze-thaw method. The transformed Gv3101 cells were syringe-infiltrated into the abaxial side of near-fully expanded leaves of 5- to 6-week-old tobacco (*Nicotiana benthamiana*) plants, as described by Liu et al. (2016). The tonoplast marker (tandem-pore K^+^ channel, TPK1) and endoplasmic reticulum (ER) marker (auxin efflux carrier family protein, PIN5) (Voelker et al., 2006; Mravec et al., 2009), fused to the red fluorescent protein (mKATE), were used for co-localization with *GmMTP8.1-GFP* or empty vector. An empty vector with GFP alone was used as a control. After 3 days of cultivation, the GFP fluorescence in epidermal cells on the abaxial leaf side was imaged using a Zeiss LSM7 DUO (Zeiss, Germany) confocal microscope. Green fluorescence was stimulated at 488 nm and detected with filter sets at 500–530 nm. The red fluorescence was excited at 561 nm, and emission was captured at 580–630 nm.

### Yeast Transformation and Metal Tolerance Analysis

Five *Saccharomyces cerevisiae* mutants, namely, the Mn-sensitive mutant Δ*pmr1* (MATa; his3Δ1; leu2Δ0; met15Δ0; ura3Δ0; YGL167c::kanMX4) defective in Mn transport into the Golgi apparatus; Zn-sensitive mutant Δ*zrc1* (MATa; ura3Δ0; leu2Δ0; his3Δ1; met15Δ0; YMR243c::kanMX4) defective in sequestering Zn into the vacuole; Cu-sensitive mutant Δ*cup2* (MATa; his3Δ1; leu2Δ0; met15Δ0; ura3Δ0; YGL166w::kanMX4) defective in a Cu-binding transcription factor for the activation of the metallothionein genes; cadmium (Cd)-sensitive mutant Δ*ycf1* (MATa; his3Δ1; leu2Δ0; met15Δ0; ura3Δ0; YDR135c::kanMX4) defective in vacuolar Cd sequestration; and cobalt (Co)-sensitive mutant Δ*cot1* (MATa; his3Δ1; leu2Δ0; met15Δ0; ura3Δ0; YOR316c::kanMX4) defective in Co efflux into the vacuole, were purchased from Euroscarf.^6^ All strains are isogenic to wild-type BY4741 (MATa; his3D1; leu2D0; met15D0; ura3D0) and are able to grow under the same conditions.

To contract yeast expression plasmid, the ORF of *GmMTP8.1* was amplified from root cDNA using *GmMTP8.1-pYES2-F/R* primers (Supplementary Table 1). The PCR product was digested with *Bam*HI and *Eco*RI, and was then inserted in the same sites of the yeast expression vector *pYES2* (Invitrogen, United States). After that, the *pYES2-GmMTP8.1* construct and *pYES2* empty vector were transformed separately into yeast cells using the LiOAc/PEG method (Gietz and Schiestl, 2007). For metal complementation analysis, yeast transformants were pre-cultured in a liquid synthetic complete medium containing a yeast nitrogen base, amino acids without uracil, and glucose (SC-U/Glu) at 30°C until the optical density (OD) at 600 nm reached a value of 0.6. Pre-cultured cells were diluted to an OD600 of 0.2, and 10-μl aliquots of dilutions (OD at 600 nm of 0.02, 0.002, and 0.0002) were spotted onto induction plates containing 2% (w) Gal, 1% (w/v) raffinose, 0.67% (w/v) yeast nitrogen base without amino acids, 0.1% (w/v) amino acids without uracil, and 2% (w/v) agar, according to Chen et al. (2015). The induction plates were added with 5 mM MnSO_4_, 15 mM ZnSO_4_, 100 μM CuSO_4_, 50 μM CdCl_2_, and 2 mM CoCl_2_ for metal complementation analyses of the yeast mutants Δ*pmr1*, Δ*zrc1*, Δ*cup2*, Δ*ycf1*, and Δ*cot1*, respectively. A control drop assay was performed on the same solid medium without the addition of metals. After incubation at 30°C for 2 days in the dark, the plates were photographed.

### Functional Analysis of *GmMTP8.1* in Arabidopsis

To construct the *pYLRNAi-GmMTP8.1* plasmid, the ORF of *GmMTP8.1* was amplified by PCR from root cDNA using *GmMTP8.1-OE-F/R* primers (Supplementary Table 1). The amplified product was digested with *Bam*HI and *Mlu*I, and subcloned further into the same site of the *pYLRNAi* vector with the *hygromycin phosphotransferase* (*HPT*) gene as the selective marker, according to Liang et al. (2010). The *pYLRNAi-GmMTP8.1* construct was transformed into *A. tumefaciens* strain Gv3101 using the freeze-thaw method. The transformed Gv3101 cells harboring the *pYLRNAi-GmMTP8.1* construct were grown further in a yeast extract peptone (YEP) medium overnight at 28°C until OD600 reached 1 before transformation. Four-week-old Columbia Arabidopsis ecotype (wild type) was used for *Agrobacterium*-mediated transformation with the floral dip method, as described by Clough and Bent (1998). Seeds of the putative transformed Arabidopsis were selected in a half-strength Murashige and Skoog (MS) solid medium containing 20 mg/L hygromycin for two generations. More than 10 T2 transgenic Arabidopsis lines were resistant to hygromycin with a 3:1 ratio. Finally, two homozygous T3 transgenic lines overexpressing *GmMTP8.1* were selected based on both qRT-PCR using *GmMTP8.1-RT-F/R* primers (Supplementary Table 1) and Western blot analysis using an anti-HPT antibody.

To assess the effects of excess Mn on the growth of Arabidopsis, seeds of the wild-type Arabidopsis and two *GmMTP8.1*-overexpression lines (OE1/2) were surface-sterilized and then germinated in an MS solid medium. Arabidopsis plants were grown in a growth chamber under conditions of 16-h light at 23°C/8-h dark at 20°C, 150 μmol m^–2^ s^–1^ photon flux density, and 70% relative humidity. After 7 days of normal growth, uniform seedlings with about 2-cm-long tap root were selected and transplanted into a fresh MS solid medium supplied with 0.1, 2 or 4 mM MnSO_4_, as described by Chen et al. (2015). Arabidopsis plants treated with 0.1 mM MnSO_4_ were set as the control. After 7 days of Mn treatments, the plants were thoroughly rinsed with 10 mM EDTA and dH_2_O. Shoots, roots, and whole plants were harvested separately to determine fresh weight and Mn concentration.

In addition, after 7 days of germination in the MS solid medium as described above, The Arabidopsis plants were transplanted into a hydroponic nutrient solution containing 0.25 mM Ca(NO_3_)_2_, 1 mM KH_2_PO_4_, 0.5 mM KNO_3_, 1 mM MgSO_4_, 50 μM Fe(III)-Na-EDTA, 50 μM H_3_BO_3_, 18 μM MnSO_4_, 1 μM CuSO_4_, 10 μM ZnSO_4_, and 0.02 μM MoO_4_Na_2_ for 14 days, according to Cailliatte et al. (2010). Subsequently, 21-day-old Arabidopsis plants were transplanted into a nutrient solution containing 18 and 400 μM MnSO_4_ as control and excess Mn treatments, respectively. After 7 days of Mn treatments, the plants were rinsed with 10 mM EDTA and dH_2_O. Shoots and roots were harvested separately to determine fresh weight and Mn concentration. All the treatments included three biological replicates.

### Analysis of Manganese Concentration

The plant samples were oven dried at 105°C for 30 min and dried further at 75°C for 5 days. Dry samples were ground into powder before Mn assay. Approximately 0.07 g of the dry samples was thoroughly burned to ash at 600°C for 10 h in a muffle furnace, according to Chen Z. et al. (2016). After cooling to room temperature, the ash sample was mixed with 7 ml of 100 mM HCl. The mixture was fully dissolved by continuous shaking for 2 days. The solution was then used to analyze Mn concentration *via* atomic absorption spectroscopy. A certified reference material, GBW07603 (bush branches and leaves) approved by Institute of Geophysical and Geochemical Exploration, Chinese Academy of Geological Sciences, China, was used to validate the extraction and measurement. Mn concentration was calculated by comparison with a standard curve.

### Western Blot Analysis

Proteins from wild-type and *GmMTP8.1*-overexpression Arabidopsis (OE1/2) were extracted separately using an extraction buffer containing 100 mM Tris–HCl (pH 8), 0.5% (w/v) PVPP, 1 mM EDTA, and 1 mM PMSF. After centrifugation at 12,000 × *g* for 15 min at 4°C, supernatants were collected. Thirty μg of extracted protein was resolved by 12% SDS-PAGE and electrophoretically transferred further to a polyvinylidene difluoride (PVDF) membrane (Merck Millipore, United States) through a Trans-Blot system (Bio-Rad, United States), according to Liang et al. (2010). After that, the PVDF membrane was incubated in a 10-mM Tris–HCl buffer (pH 8) containing 5% (w/v) non-fat dry milk and 150 mM NaCl for 12 h. The PVDF membrane was then washed three times with a washing solution containing 10 mM Tris–HCl (pH 8), 150 mM NaCl, and 0.1% (v/v) Tween 20. Blotting of the PVDF membrane was performed by incubation with an anti-HPT primary antibody (1:1,000 dilution) in a blotting buffer containing 10 mM Tris–HCl (pH 8), 150 mM NaCl, 0.1% (v/v) Tween 20, and 3% (w/v) non-fat dry milk for 1 h, followed by three washes with a washing buffer. An alkaline phosphatase-tagged secondary antibody was then added into the fresh blotting buffer, where the PVDF membrane was incubated for 1 h. After three washes with the washing buffer, the band of target protein in the PVDF membrane was observed after alkaline phosphatase reaction.

### Statistical Analyses

The SPSS program v13.0 (SPSS Institute, United States) was used to perform one-way ANOVA and Student’s *t*-test analyses.

## Results

### Soybean Growth Inhibited by Manganese Toxicity

**FIGURE 1:**
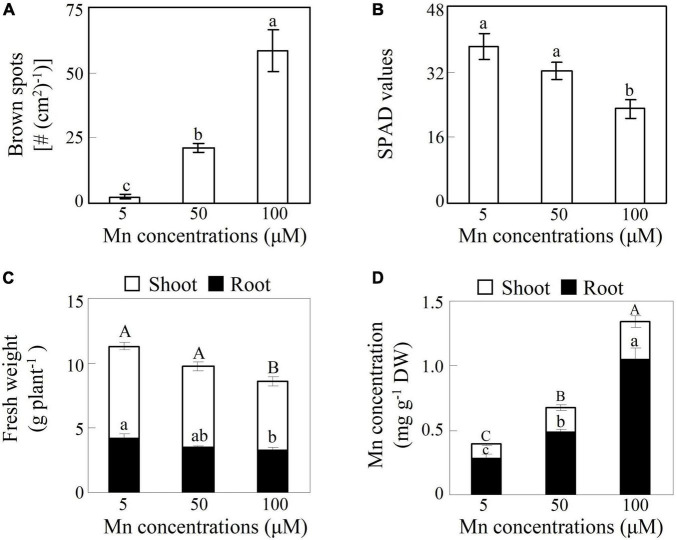
Effects of excess manganese (Mn) treatment on soybean growth. **(A)** Number of brown spots. **(B)** Soil plant analysis development (SPAD) values. **(C)** Fresh weight. **(D)** Mn concentrations. Fourteen-day-old soybean seedlings were treated with 5, 50, and 100 μM MnSO_4_ for 7 days. Five μM MnSO_4_ was used as the control. Chlorophyll levels were detected with SPAD-502 meter. Each bar represents the means of four independent replicates with standard error. Different lower case letters indicate significant differences at *P* < 0.05 among Mn treatments in panels **(A,B)**. Different upper case or lower case letters indicate significant differences at *P* < 0.05 among Mn treatments in shoot or root in panels **(C,D)**. White and black bars represent shoot and root, respectively.

To investigate the toxic effects of Mn on soybean growth, 14-day-old soybean seedlings were exposed to different Mn treatments for 7 days. The results showed that excess Mn treatments (50 and 100 μM) resulted in the generation of brown spots on mature leaves compared to the control (5 μM Mn) (Figure 1A). Moreover, the number of brown spots with the 100 μM Mn treatment was 1.8-fold higher than that with the 50-μM Mn treatment (Figure 1A). In contrast, chlorophyll levels, indicated by SPAD values in leaves, were decreased by 15.7 and 39.9% with the 50- and 100-μM Mn treatments, respectively, compared to the control (Figure 1B). Although both shoot and root fresh weights were unaffected by the 50-μM Mn treatment, they were significantly decreased by more than 24% with100 μM Mn compared to the control (Figure 1C). In addition, increases in Mn concentrations were observed in shoots and roots of soybean under excess Mn treatments. Mn concentrations in shoots at 50 and 100 μM Mn were 79.8–175.3% higher than 5-μM Mn treatments, while Mn levels in roots at 50 and 100 μM Mn were 70.7–269.1% higher than the controls (Figure 1D). Furthermore, Mn concentrations in roots were more than 155% higher than in shoots under excess Mn stress (50 and 100 μM) (Figure 1D).

### Identification of *GmMTP* Genes in Soybean

**FIGURE 2:**
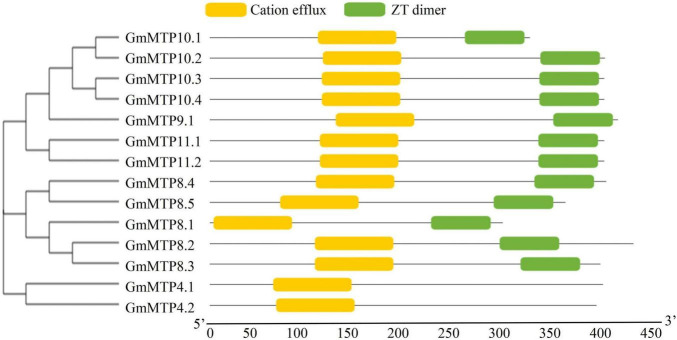
Conserved motifs in GmMTPs. Yellow and green boxes indicate cation efflux and ZT dimer motifs, respectively. The neighbor-joining phylogenetic tree of GmMTPs was constructed using MEGA4.1.

In this study, a total of 14 putative *GmMTP* genes were identified in the soybean genome. The 14 *GmMTP* genes were named from *GmMTP4.1* to *GmMTP11.2* based on the phylogenetic relationship and sequence identity between GmMTP and Arabidopsis AtMTPs (Supplementary Table 2). General information for the 14 *GmMTP* members is summarized in Supplementary Table 2. Most of the GmMTP members contained three to six conserved TMDs, except GmMTP10.1, which harbored only two TMDs (Supplementary Table 2). The 14 *GmMTP* genes can be classified into three distinct groups according to their sequence structure features (Supplementary Figure 1). Furthermore, most GmMTPs contained both the cation efflux (PF01545) and zinc transporter (ZT) dimer (PF16916) conserved motifs, except GmMTP4.1 and GmMTP4.2, which only contained the cation efflux motif (Figure 2 and Supplementary Table 3).

**FIGURE 3:**
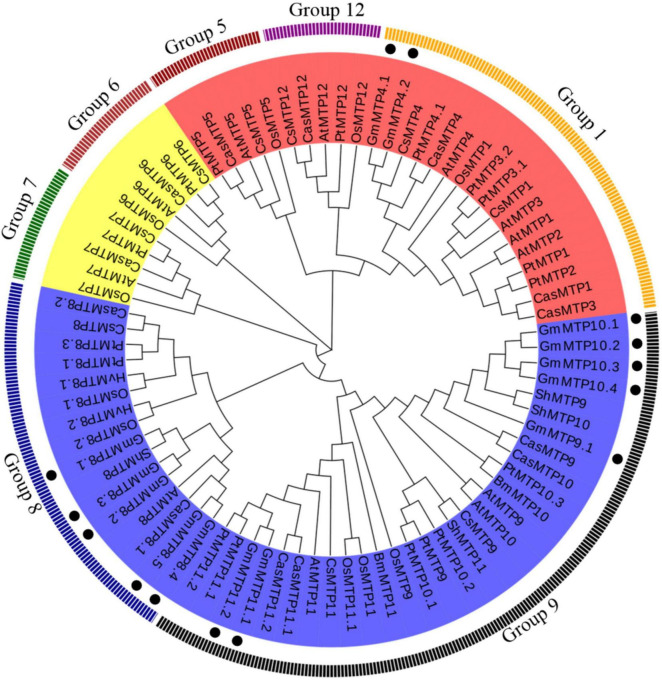
Phylogenetic analysis of metal tolerance proteins (MTPs) in soybean and other plants. The phylogenetic tree was constructed using the MEGA4.1 program. Except for *CasMTPs* derived from tea plant (*Camellia sinensis*), the first two letters of each protein represent the abbreviated species name. At: *Arabidopsis thaliana*, Os: *Oryza sativa*, Gm: *Glycine max*, Cs: *Cucumis sativus*, Hv: *Hordeum vulgare*, Pt: *Populus trichocarpa*, Sh: *Stylosanthes hamata*, and Bm: *Beta vulgaris*. ssp. *maritima*. MTPs from various plant species are divided into the Zn-CDF, Fe/Zn-CDF, and Mn-CDF clusters, which are highlighted in red, yellow, and blue, respectively. Plant MTPs can be further classified into seven subgroups. The solid circles indicate MTPs from soybean.

Phylogenetic analysis showed that plant MTPs can be classified into three distinct clusters, namely, Zn-CDF, Zn/Fe-CDF, and Mn-CDF, which were further divided into seven subgroups (Figure 3). Of the seven subgroups, group 9 had the largest number of plant MTPs, followed by groups 1 and 8. A total of 12 GmMTPs were classified into groups 8 and 9, both of which belonged to the Mn-CDF cluster (Figure 3). Among them, five GmMTPs (GmMTP8.1, GmMTP8.2, GmMTP8.3, GmMTP8.4, and GmMTP8.5) clustered with ShMTP8 from *S. hamata*, AtMTP8 from Arabidopsis, OsMTP8.1/8.2 from rice, CsMTP8 from cucumber, HvMTP8.1/8.2 from barley (*Hordeum vulgare*), and CasMTP8.1/8.2 from tea plant (*Camellia sinensis*) in group 8. Seven GmMTPs, namely, GmMTP9.1, GmMTP10.1, GmMTP10.2, GmMTP10.3, GmMTP10.4, GmMTP11.1, and GmMTP11.2, together with OsMTP9/11/11.1 from rice, CsMTP9 from cucumber, AtMTP11 from Arabidopsis, PtMTP11.1/11.2 from poplar, and BmMTP10/11 from *B. vulgaris* ssp. *maritima*, were divided into group 9 (Figure 3). In addition, GmMTP4.1 and GmMTP4.2 were classified into group 1 within the Zn-CDF cluster (Figure 3). Interestingly, none of the GmMTP members were classified into the Fe/Zn-CDF cluster (Figure 3).

### Expression Analysis of *GmMTP* Genes

**FIGURE 4:**
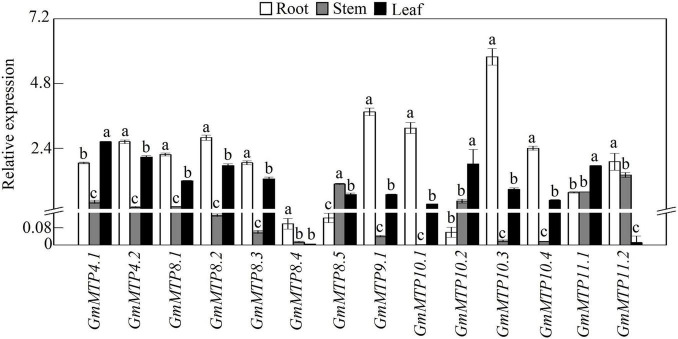
Expressions of *GmMTPs* in leaf, stem, and root of soybean. After seeds were germinated for 7 days, the seedlings were transplanted into a full-strength Hoagland solution. Leaf, stem, and root were harvested separately after 14 days of growth for gene expression analysis. Each bar represents the mean value of three independent replicates with standard error. White, gray, and black bars represent root, stem, and leaf, respectively. Different lower case letters indicate significant differences at *P* < 0.05 in the same tissue.

We subsequently analyzed the expression patterns of *GmMTP* genes in leaves, stems, and roots of 21-day-old soybean seedlings under normal growth conditions. The results showed that *GmMTPs* exhibited differential expressions in various tissues of soybean (Figure 4). Among them, three *GmMTPs* (*GmMTP4.1*, *GmMTP10.2*, and *GmMTP11.1*) showed highest expression in leaves, while 10 *GmMTPs* (*GmMTP4.2*, *GmMTP8.1*, *GmMTP8.2*, *GmMTP8.3*, *GmMTP8.4*, *GmMTP9.1*, *GmMTP10.1*, *GmMTP10.3*, *GmMTP10.4*, and *GmMTP11.2*) were mainly expressed in roots (Figure 4). Interestingly, most of the *GmMTP* genes exhibited low expressions in stems, except *GmMTP8.5* with the highest expression in stems compared to leaves or roots (Figure 4). Furthermore, the transcript of *GmMTP4.1* in leaves was higher than that of the other *GmMTPs*, while *GmMTP10.3* exhibited the highest expression in roots compared to the other *GmMTPs* (Figure 4).

**FIGURE 5:**
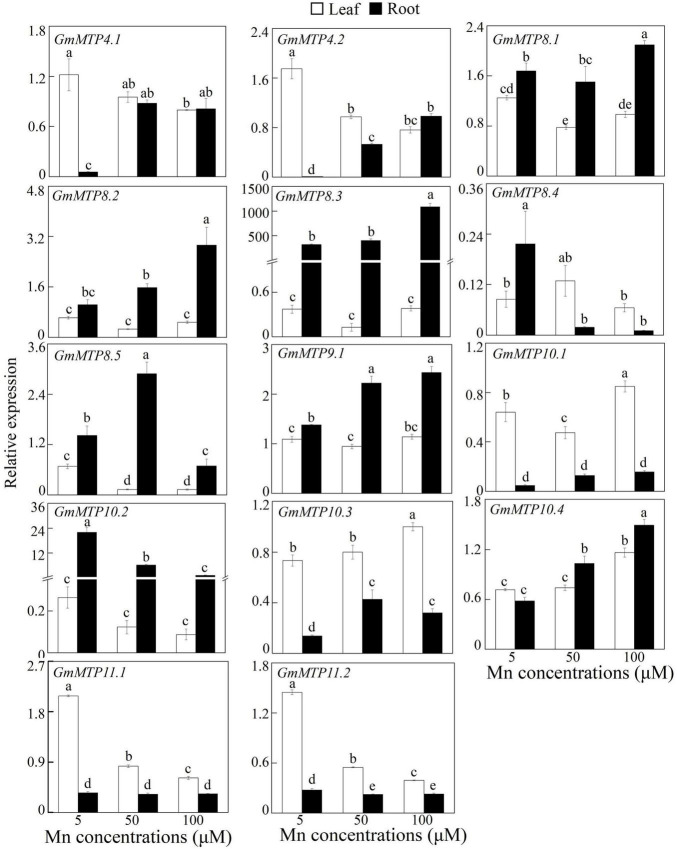
Expressions of *GmMTPs* in leaf and root of soybean under excess Mn treatments. Fourteen-day-old soybean seedlings were transferred into a nutrient solution containing 5, 50, or 100 μM MnSO_4_. Five μM MnSO_4_ was used as the control. After 7 days of Mn treatment, leaves and roots were harvested separately for gene expression analysis. Each bar represents the mean value of three independent replicates with standard error. White and black bars represent leaf and root, respectively. Different lower case letters indicate significant differences at *P* < 0.05.

Expressions of the *GmMTPs* in leaves and roots in response to Mn toxicity were investigated. As shown in Figure 5, all *GmMTPs* responded to excess Mn in leaves and/or roots, and the number of *GmMTPs* regulated by Mn stress in roots (12 *GmMTPs*) was higher than those in leaves (9 *GmMTPs*). For example, two *GmMTPs* (*GmMTP10.3* and *10.4*) were enhanced and six *GmMTPs* (*GmMTP4.1*, *GmMTP4.2*, *GmMTP8.1*, *GmMTP8.5*, *GmMTP11.1*, and *GmMTP11.2*) were suppressed in leaves by the 50- and/or 100-μM Mn treatments. In contrast, most *GmMTPs* (*GmMTP4.1*, *GmMTP4.2*, *GmMTP8.1*, *GmMTP8.2*, *GmMTP8.3*, *GmMTP8.5*, *GmMTP9.1*, *GmMTP10.3*, and *GmMTP10.4*) were enhanced and only three *GmMTPs* (*GmMTP8.4*, *GmMTP10.2*, and *GmMTP11.2*) were suppressed in roots by the 50- and/or 100-μM Mn treatments (Figure 5). Interestingly, the transcripts of *GmMTP10.3*, *GmMTP10.4*, and *GmMTP11.2* regulated by Mn in roots were similar to those in leaves (Figure 5).

Similar to the regulation by excess Mn stress, the number of *GmMTPs* regulated by excess Fe, Zn, and Cu stresses in roots was higher than those in leaves (Supplementary Figure 2). For example, in roots, a total of 13, 12, and 11 *GmMTPs* were regulated by Fe, Zn, and Cu stresses, respectively. In leaves, 7, 9, and 9 *GmMTPs* were found to be regulated by Fe, Zn, and Cu stresses, respectively. Furthermore, most of the *GmMTP* genes were regulated by at least one metal stress in both leaves and roots, except *GmMTP9.1*, *GmMTP10.4*, and *GmMTP11.2* that were not responsive in leaves to any of the three tested metals (Supplementary Figure 2). Interestingly, five *GmMTPs* (*GmMTP4.1*, *GmMTP4.2*, *GmMTP8.3*, *GmMTP8.4*, and *GmMTP10.2*) were simultaneously suppressed, and three *GmMTPs* (*GmMTP4.1*, *GmMT8.5*, and *GmMT10.1*) were concurrently enhanced in leaves and roots by all the three metals, respectively (Supplementary Figure 2).

### Subcellular Localization of GmMTP8.1

In this study, GmMTP8.1, a member of group 8 within the Mn-CDF cluster (Figure 3), displayed highest similarity with ShMTP8 in *S. hamata* that has been well-characterized for its involvement in Mn detoxification by the sequestration of Mn into vacuoles, was further selected to dissect its function in Mn detoxification. The GmMTP8.1 protein includes 294 amino acid residues (33.1 kDa) and possesses common features of Mn-CDF transporters, such as four putative TMDs, cation efflux, and ZT dimer domains (Figure 2 and Supplementary Table 2). The consensus sequence DxxxD (x = any amino acid) localizes to the first TMD and the cytosolic loop of GmMTP8.1 (Supplementary Figure 3). In addition, based on amino acid sequence comparisons, GmMTP8.1 shares a high degree of homology identity (90.8%) with ShMTP8 in *S. hamata*, and shares 83.3% identity with HvMTP8.1 in barley, 83% identity with OsMTP8.1 in rice, 83% identity with CsMTP8 in cucumber, and 81% identity with AtMTP8 in Arabidopsis, which were the representative MTP8-like proteins included in group 8 (Supplementary Figures 3–5). This suggests that the role of GmMTP8.1 is similar to that of the MTP homologs.

**FIGURE 6:**
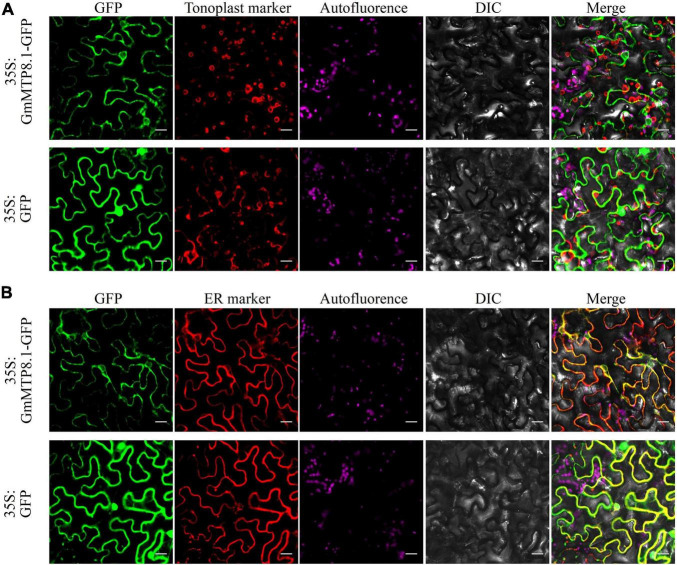
Subcellular localization of GmMTP8.1 in tobacco leaf lower epidermal cells. The images are labeled to show the *35S:GFP* and *35S:GFP-GmMTP8.1* constructs. *35S:GFP-GmMTP8.1* was co-expressed with the **(A)** tonoplast marker TPK1 and **(B)** endoplasmic reticulum marker PIN5 fused with red fluorescence protein (mKATE) in tobacco leaf epidermal cells. green fluorescent protein (GFP) fluorescence, red fluorescent protein (RFP) fluorescence, chloroplast autofluorescence, bright field images, and merged images are displayed from left to right. Fluorescence was observed by confocal microscopy. Scale bar is 20 μm.

We further analyzed the subcellular localization of GmMTP8.1 in tobacco epidermis cells. The results showed that the GFP signal of GmMTP8.1 was found to be co-localized with that of the ER marker but not localized in the tonoplast of tobacco leaf epidermal cells, whereas the fluorescence of cells transformed with GFP alone was observed in whole cells, such as plasma membrane, cytoplasm, and nucleus (Figures 6A,B). Thus, these results suggest that GmMTP8.1 is localized to the ER.

### Metal Transport Activity of GmMTP8.1 in Yeast Cells

**FIGURE 7:**
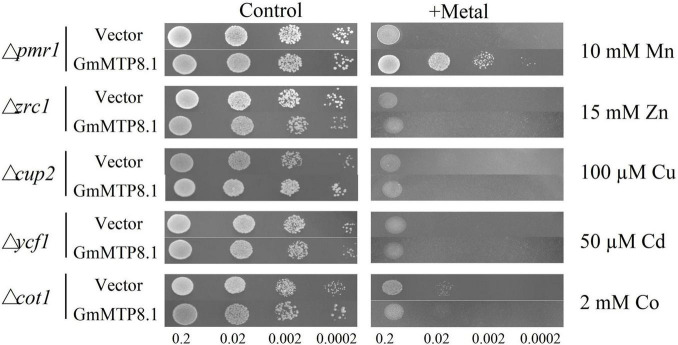
Heterologous expression of *GmMTP8.1* in different yeast mutants. The yeast mutants Δ*pmr1*, Δ*zrc1*, Δ*cup2*, Δycf1, and Δ*cot1* harboring the *pYES2* empty vector or the *pYES2-GmMTP8.1* construct were used. Yeast cells (10 μl) with OD600 of 0.2 and four serial dilutions (10-fold) were spotted in SC-U/Gal medium added with or without 5 mM MnSO_4_, 15 mM ZnSO_4_, 100 μM CuSO_4_, 50 μM CdCl_2_, and 2 mM CoCl_2_ for 2 day at 30°C.

A complementation assay of yeast mutants was further conducted to investigate the metal transport activity of GmMTP8.1, which was heterologously expressed in five yeast mutants that are defective in transport activity of various metals, namely, Mn (Δ*pmr1*), Zn (Δ*zrc1*), Cu (Δ*cup2*), Cd (Δ*ycf1*), and Co (Δ*cot1*). The results showed that the yeast mutants carrying either the *pYES2* empty vector or the *pYES2-GmMTP8.1* construct grew similarly in the control medium (Figure 7). However, the growth of the five yeast mutants transformed with the *pYES2* empty vector was inhibited in the medium containing high or toxic metal levels (Figure 7). Furthermore, although the growth of the yeast mutants Δ*zrc1*, Δ*cup2*, Δ*ycf1*, and Δ*cot1*, transformed with the *pYES2-GmMTP8.1* construct, was suppressed in the corresponding metal treatments, *GmMTP8.1* expression rescued the sensitivities to excess Mn in the Δ*pmr1* mutant (Figure 7), suggesting that *GmMTP8.1* is a specific Mn transporter.

### Overexpression of *GmMTP8.1* Enhanced Manganese Tolerance and Reduced Manganese Accumulation in Arabidopsis

**FIGURE 8:**
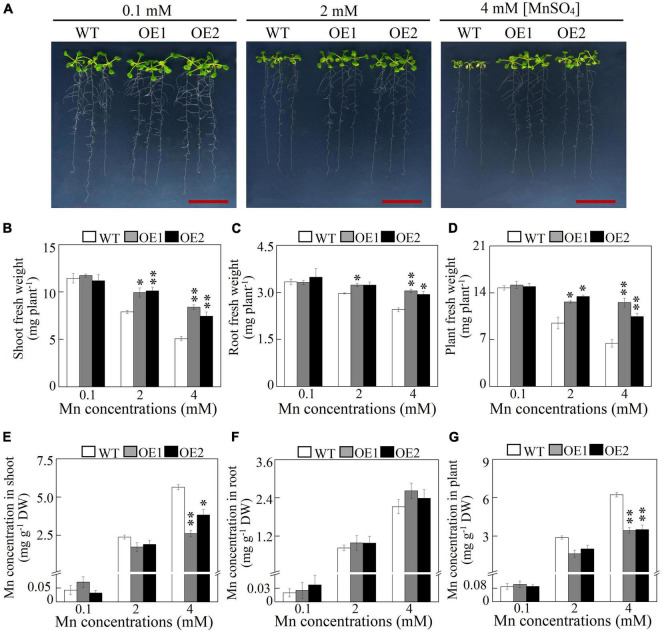
Growth and Mn concentration of Arabidopsis overexpressing *GmMTP8.1*. **(A)** Phenotype of Arabidopsis grown with different Mn treatments. **(B)** Shoot fresh weight. **(C)** Root fresh weight. **(D)** Plant fresh weight. **(E)** Mn concentration in shoot. **(F)** Mn concentration in root. **(G)** Mn concentration in plant. Seven-day-old Arabidopsis seedlings were transferred to a solid Murashige and Skoog (MS) medium containing 0.1, 2, or 4 mM MnSO_4_. Arabidopsis plants treated with 0.1 mM MnSO_4_ were set as the control. After 7 days of Mn treatment, fresh weight and Mn concentration were determined. WT represents the wild type Arabidopsis. OE1 and OE2 are two transgenic lines overexpressing *GmMTP8.1*. Each bar represents the mean value of three independent replicates with standard error. White, gray, and black bars represent WT, OE1, and OE2, respectively. Asterisks indicate significant differences between the wild type and the overexpression lines with the same Mn treatment. *, 0.01 < *P* < 0.05. **, 0.001 < *P* < 0.01. DW, dry weight. Scale bar is 2 cm.

Subsequently, transgenic Arabidopsis overexpressing *GmMTP8.1* was generated to investigate the roles of *GmMTP8.1* in Mn detoxification in the plants. Increased transcripts of *GmMTP8.1* in the overexpression lines (OE1 and OE2) compared to the wild-type (WT) were confirmed by RT-PCR analysis (Supplementary Figure 6A). Western-blot analysis indicated further that the GmMTP8.1 protein was expressed in transgenic Arabidopsis (Supplementary Figure 6B). WT and transgenic Arabidopsis were exposed to different levels of Mn, from 0.1 to 4 mM, in an MS solid medium for 7 days. As shown in Figure 8A, no difference in Arabidopsis growth was observed between the *GmMTP8.1* overexpression lines (OE1 and OE2) and WT under control conditions containing 0.1 mM Mn. Although excess Mn (2 mM and 4 mM Mn) applications inhibited the growth of both overexpression lines and WT, the overexpression lines exhibited a higher level of tolerance to excess Mn than WT, especially with the 4-mM Mn treatment (Figure 8A). Shoot, root, and whole plant fresh weight of the overexpression lines were 46.6-65.2, 19.7-24.5, and 62.2-94.8%, respectively, higher than those of WT with the 4-mM Mn treatment (Figures 8B-D). In contrast, the overexpression of *GmMTP8.1* resulted in significantly decreased Mn accumulation in shoots and whole plants of transgenic Arabidopsis exposed to the 4-mM Mn treatment, although no differences in root Mn accumulation were observed between WT and the transgenic lines (Figures 8E-G). Mn concentrations in shoots of the overexpression lines were 32.2–53.5% lower than those found in WT grown at 4 mM Mn (Figure 8E). Furthermore, total Mn concentrations in the transgenic Arabidopsis plants were 44–45% less than those in WT exposed to 4 mM Mn treatment (Figure 8G).

In addition, we also assessed the effects of excess Mn on the growth of transgenic Arabidopsis in hydroponics. After growing in a nutrient solution containing 18 or 400 μM MnSO_4_ for 7 days, the transgenic lines exhibited improved Mn tolerance compared to the WT (Supplementary Figure 7). Under the 400-μM Mn treatment, shoot fresh weight of the overexpression lines was more than 53% higher than that of the WT, although root fresh weight was similar between the transgenic lines and WT (Supplementary Figure 7). Consistent with Mn concentration in Arabidopsis grown in the MS solid medium, a low level of Mn concentration in shoots but not roots was observed in transgenic Arabidopsis compared to the WT, exposed to the 400-μM Mn treatment (Supplementary Figure 7). These results, together, suggest that the overexpression of *GmMTP8.1* confers Mn tolerance in Arabidopsis mainly by decreasing accumulation of Mn.

### Manganese Transporters Are Upregulated by Overexpression of *GmMTP8.1* in Arabidopsis

**FIGURE 9:**
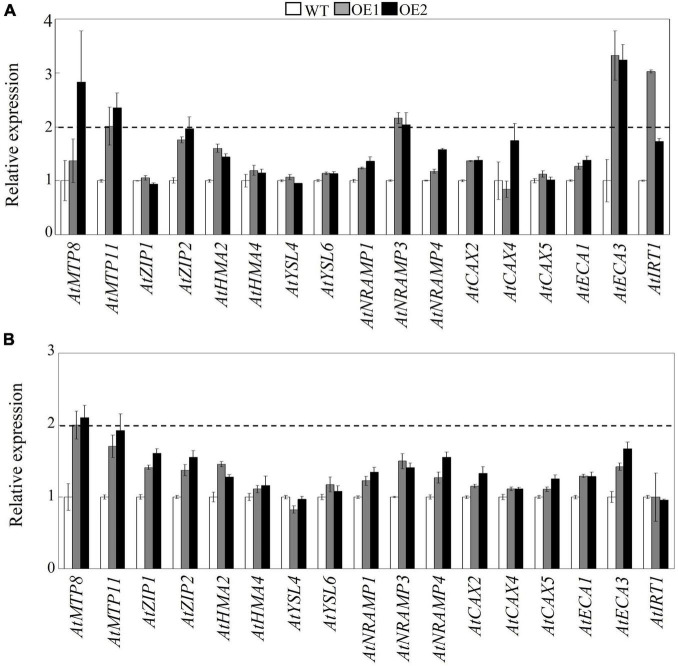
Effects of *GmMTP8.1* overexpression on the transcript levels of Mn transporter genes in Arabidopsis. **(A)** Gene expression in leaves. **(B)** Gene expression in roots. Seven-day-old seedlings were transferred to a solid MS medium containing 4 mM MnSO_4_ for 7 days. Leaves and roots were sampled for RNA extraction and real-time (RT)-polymerase chain reaction (PCR) analysis. WT represents the wild type Arabidopsis. OE1 and OE2 are two transgenic lines overexpressing *GmMTP8.1*. Each bar represents the mean value of three independent replicates with standard error. White, gray, and black bars represent WT, OE1, and OE2, respectively. The relative expression level of gene of more than 2-fold was regarded as significant difference between the wild type and the overexpression lines.

To investigate the mechanisms involved in decreased Mn in the shoots but unchanged level of Mn in the roots of transgenic Arabidopsis, the transcript levels of 17 Mn transporter genes were determined in transgenic Arabidopsis overexpressing *GmMTP8.1*. These Mn transporters included genes encoding metal tolerance proteins (*AtMTP8/11*), calcium exchangers (*AtCAX2/4/5*), ER-type calcium (Ca^2+^)-ATPases (*AtECA1/3*), iron-regulated transporter (*AtIRT1*), natural resistance-associated macrophage proteins (*AtNRAMP1/3/4*), zinc transporters (*AtZIP1/2*), yellow stripe-like proteins (*AtYSL4/6*), and heavy metal ATPases (*AtHMA2/4*). The qRT-PCR analysis showed that the overexpression of *GmMTP8.1* led to a significant increase (>2-fold) in the transcripts of *AtMTP11*, *AtNRAMP3*, and *AtECA3* in leaves of the two transgenic lines (Figure 9). However, among the tested genes, only *AtMTP8* exhibited about 2.1-fold induction in roots of the transgenic Arabidopsis (Figure 9). The upregulation of these Mn transporters might contribute to the regulation of Mn homeostasis and tolerance in transgenic Arabidopsis.

## Discussion

Manganese is a trace element that can cause phytotoxicity when present in excess (Millaleo et al., 2010; Li et al., 2019). Excess Mn disrupts various physiological processes in plants. For example, Mn toxicity causes oxidative stress and lipid peroxidation in cowpea (*Vigna unguiculata*) and cucumber (Fecht-Christoffers et al., 2006; Dragišić Maksimović et al., 2012), inhibits chlorophyll biosynthesis and photosynthesis in *Stylosanthes guianensis* (Liu P. et al., 2019; Jia et al., 2020), and disturbs the homeostasis of other nutrients in polish wheat (*Triticum polonicum*) (Sheng et al., 2015). In this study, excess Mn treatments led to increase in Mn levels in soybean, which was accompanied by increased number of brown spots and decreased chlorophyll concentrations, ultimately reducing plant fresh weight of the 14-day-old soybean seedlings (Figure 1). Similar results have been found in previous studies where soybean growth was inhibited by Mn toxicity (Chen Z. et al., 2016; Santos et al., 2017; Liu et al., 2020), although different growth stages of soybean were applied. Furthermore, important roles of antioxidant systems, such as antioxidant enzymes and non-enzymatic components, have been suggested in the response of soybean to Mn toxicity (Chen Z. et al., 2016; Santos et al., 2017; Liu et al., 2020). Although some advances have been made in investigating the response of soybean to Mn toxicity, its potential molecular mechanisms involved in Mn tolerance have not been elucidated.

Plant MTPs have been documented to play critical roles in Mn detoxification by Mn sequestration or efflux from plant cells (Li et al., 2019, 2021; Alejandro et al., 2020). In order to investigate the involvement of *GmMTPs* in the tolerance of soybean to Mn toxicity, we first identified 14 *GmMTP* genes in the soybean genome. Most of them were found to contain the conserved cation efflux and ZT dimer motifs (Figure 2). These motifs are reported to be essential for metal transport activities of MTPs (Montanini et al., 2007; Kolaj-Robin et al., 2015; Chen Z. et al., 2016). Cumulative studies demonstrated conserved functions of the MTP homologues in Mn detoxification in various plant species, suggesting similar roles for GmMTPs in Mn tolerance of soybean. According to the phylogenetic analysis, five GmMTPs (GmMTP8.1, GmMTP8.2, GmMTP8.3, GmMTP8.4, and GmMTP8.5) were classified into group 8, and seven GmMTPs (GmMTP9.1, GmMTP10.1, GmMTP10.2, GmMTP10.3, GmMTP10.4, GmMTP11.1, and GmMTP11.2) were classified into group 9 (Figure 3). Group 8 contained representative MTPs from other plant species implicated in Mn detoxification mainly through sequestration of Mn by transport into vacuoles, such as ShMTP8 from *S. hamata*, OsMTP8.1/8.2 from rice, CsMTP8 from cucumber (Delhaize et al., 2003; Chen et al., 2013; Migocka et al., 2014; Takemoto et al., 2017). In addition, two MTP8-like proteins from the tea plant have recently been reported to participate in Mn detoxification *via* efflux of Mn from plant cells (Li et al., 2017; Zhang et al., 2020). On the other hand, group 9 also included MTP9/10/11 homologues from Arabidopsis, rice, poplar, and *B. vulgaris* with similar functions in sequestration of Mn into endomembrane compartments or export of Mn out of cells (Peiter et al., 2007; Erbasol et al., 2013; Tsunemitsu et al., 2018a).

Subsequent expression analysis showed that nine *GmMTPs* (*GmMTP4.1*, *GmMTP4.2*, *GmMTP8.1*, *GmMTP8.2*, *GmMTP8.3*, *GmMTP8.5*, *GmMTP9.1*, *GmMTP10.3*, and *GmMTP10.4*) and two *GmMTPs* (*GmMTP10.3* and *GmMTP10.4*) were enhanced by excess Mn in roots and leaves, respectively (Figure 5). Similarly, a set of *MTP* homologues engaged in Mn detoxification are also upregulated by excess Mn in leaves or roots, such as *AtMTP8* in Arabidopsis (Eroglu et al., 2016), *CsMTP8/9* in cucumber (Migocka et al., 2014, 2015), *HvMTP8.1* in barley (Pedas et al., 2014), *CasMTP8.1/8.2* in tea plant (Li et al., 2017; Zhang et al., 2020), and *BmMTP10* in *B. vulgaris* (Erbasol et al., 2013). However, the transcripts of *AtMTP11* in Arabidopsis and *OsMTP8.1/8.2* in rice, which are also involved in Mn detoxification (Delhaize et al., 2007; Peiter et al., 2007; Takemoto et al., 2017), are unaffected by excess Mn levels. It is estimated that functions of some of *MTP* members might not depend on external Mn at transcriptional levels but at post-transcriptional levels. For example, although the transcript of *OsMTP8.1* in rice is not affected by excess Mn, the accumulation of OsMTP8.1 protein is significantly increased under excess Mn conditions, contributing to Mn tolerance (Chen et al., 2013).

In this study, five MTP8-like members (GmMTP8.1 to GmMTP8.5) in soybean that clustered closely showed similar structures and homology identities (Figure 2 and Supplementary Figures 1, 5), which is consistent with the results of phylogenetic analysis (Figure 3). However, GmMTP8.1 to GmMTP8.5 possessed different numbers of exons and introns, and GmMTP8.1 had the lowest molecular size (Supplementary Table 2). Furthermore, variations in the expression levels of these five *GmMTP8s* were observed in leaves or roots of soybean response to different Mn levels (Figure 5). Sequence and gene expression differences among the *GmMTP8s* suggest that these genes may have distinct or redundant biological functions in Mn detoxification. Similarly, although OsMTP8.1 and OsMTP8.2 are two MTP8-like homologues in rice, the abundance of *OsMTP8.1* in shoots is regulated by external Mn levels, and *OsMTP8.2* does not respond to the Mn treatments (Chen et al., 2013; Takemoto et al., 2017). Furthermore, although the disruption of *MTP8.2* does not affect Mn tolerance in rice, the knockdown of *MTP8.2* in the *mtp8.1* mutant results in severe growth inhibition under Mn toxicity conditions (Takemoto et al., 2017), suggesting that OsMTP8.1 coordinates with OsMTP8.2 to increase Mn tolerance in rice. Thus, the five GmMTP8s might possess various functions in soybean response to Mn toxicity, which merits further verification.

A detailed functional analysis of GmMTP8.1, which shares a high degree of homology identity with ShMTP8 (90.8%), OsMTP8.1 (83%), CsMTP8 (83%), and AtMTP8 (81%) (Supplementary Figures 3–5), was performed further. GmMTP8.1 contained four TMDs, a signature sequence specific to the CDF family, and two short motifs (DSLLD and DHYFD) specific to the Mn-CDF cluster (Supplementary Figure 3). These common features are also present in the reported Mn transporters, such as ShMTP8, CsMTP8, and OsMTP8.1/8.2 (Delhaize et al., 2003; Chen et al., 2013; Migocka et al., 2014; Takemoto et al., 2017), suggesting similar roles of GmMTP8.1 in conferring Mn tolerance. It has been demonstrated that *ShMTP8* in *S. hamata* can complement the phenotype of yeast mutant Δ*pmr1* defective in Mn transport under excess Mn conditions; the overexpression of *ShMTP8* increases Mn tolerance in Arabidopsis by sequestration of Mn into vacuoles (Delhaize et al., 2003). Furthermore, in Arabidopsis, AtMTP8 is found to localize in the tonoplast, and the T-DNA insertion Arabidopsis *mtp8* mutant displays greater sensitivity to excess Mn than the wild type (Eroglu et al., 2016). Similar functions of MTP8-like proteins have also been observed in OsMTP8.1/8.2 in rice and CsMTP8 in cucumber (Chen et al., 2013; Migocka et al., 2014; Takemoto et al., 2017; Zhang et al., 2021). In this study, heterologous expression of *GmMTP8.1* led to restore the growth of the yeast mutant *pmr1* to excess Mn (Figure 7), suggesting that GmMTP8.1 is responsible for Mn transport. Furthermore, the overexpression of *GmMTP8.1* conferred Mn tolerance in transgenic Arabidopsis grown in both MS solid medium and hydroponic system (Figure 8 and Supplementary Figure 7). Therefore, GmMTP8.1 contributes to increase tolerance to Mn in soybean through regulation of Mn transport.

**FIGURE 10:**
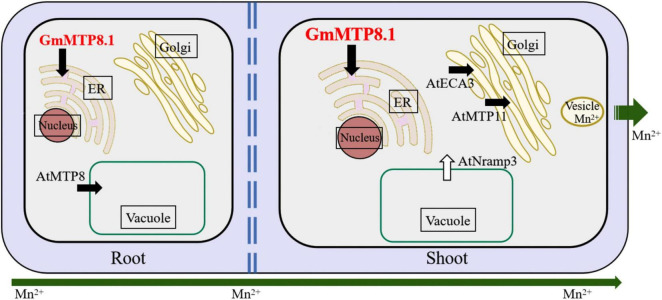
Hypothetical view of GmMTP8.1 affecting Mn tolerance in transgenic Arabidopsis. Endoplasmic reticulum (ER)-localized GmMTP8.1 is involved in Mn transport into the ER, which has probably contributed in reduction of cytosolic Mn and maintenance of Mn homeostasis in transgenic Arabidopsis overexpressing *GmMTP8.1*. Furthermore, cytosolic Mn in leaf can be transported into the Golgi apparatus by the involvement of Golgi-localized AtMTP11 and ECA3, and then sequestered into vesicles and removed from the cell *via* exocytosis, thereby decreasing cellular Mn levels in shoot. The upregulation of *AtNRAMP3* might also help to maintain Mn homeostasis in leaves of transgenic Arabidopsis. In addition, GmMTP8.1 has likely mediated Mn tolerance, along with AtMTP8, by increasing Mn sequestration into root vacuoles, conferring Mn tolerance. White and black arrows represent import into and export out of the cytosol, respectively.

Although GmMTP8.1 exhibited high similarity with the MTP8-like proteins, some functional variations are observed among these homologues. For example, the representative MTP8 members, namely, ShMTP8, CsMTP8, OsMTP8.1/8.2, and AtMTP8, are found to be localized to the tonoplast, detoxifying Mn by sequestration of Mn into the vacuoles (Delhaize et al., 2003; Chen et al., 2013; Migocka et al., 2014; Eroglu et al., 2016; Takemoto et al., 2017). However, HvMTP8.1 and HvMTP8.2 from barley are suggested to be involved in delivering Mn to the Golgi apparatus, regulating Mn homeostasis (Pedas et al., 2014). Furthermore, two MTP8 homologues from the tea plant are localized to the plasma membrane and participated in Mn efflux from plant cells (Li et al., 2017; Zhang et al., 2020). Unexpectedly, different from those identified MTP8-like proteins, GmMTP8.1 was found to be localized to the ER in tobacco leaf epidermis cells (Figure 6), suggesting that GmMTP8.1 is involved in Mn transport into the ER, which has probably contributed to reduction in cytosolic Mn and maintenance of Mn homeostasis in transgenic Arabidopsis overexpressing *GmMTP8.1* (Figure 10). Similarly, the ER-localized AtECA1 has been found to promote Arabidopsis growth under Mn toxicity conditions by pumping Mn into the ER, reducing cytosolic Mn to levels that do not disrupt other elements homeostasis (Wu et al., 2002).

In this study, less Mn accumulation was observed in shoots but not roots of transgenic Arabidopsis overexpressing *GmMTP8.1* than in those of WT under excess Mn conditions (Figure 8 and Supplementary Figure 7), suggesting that GmMTP8.1 participates in the extrusion of Mn from leaf cells. The involvement of MTP homologues in Mn efflux has been observed in several plants, such as CasMTP8.1/8.2 in tea plant, HvMTP8.1/8.2 in barley, CsMTP9 in cucumber, AtMTP11 in Arabidopsis, and OsMTP11 in rice (Peiter et al., 2007; Pedas et al., 2014; Migocka et al., 2015; Li et al., 2017; Ma et al., 2018; Zhang et al., 2020). These MTPs are generally localized to the Golgi apparatus or the plasma membrane (Pedas et al., 2014; Li et al., 2017; Zhang et al., 2020). However, ER-localized MTP8-like proteins involved in cellular Mn extrusion have not been reported, although ShMTP8 also seems to target the ER membrane in yeast cells (Delhaize et al., 2003). This study indicates the role of ER-localized GmMTP8.1 participating in the export of Mn out of the leaf cells (Figure 10). Such a cellular Mn extrusion pathway has previously been suggested for the participation of the Golgi-localized AtMTP11 in Arabidopsis and OsMTP11 in rice, in that Mn can be loaded into the *trans*-Golgi network (TGN), sequestered into vesicles, trafficked to the plasma membrane, and ultimately released to the extracellular space (Peiter et al., 2007; Ma et al., 2018). As GmMTP8.1 was localized to the ER and displayed less similarity with AtMTP11 (56.8%) and OsMTP11 (58.5%), it might indirectly participate in stimulation of the cellular extrusion of Mn. Thus, we speculated that GmMTP8.1 might influence Mn distribution and status, subsequently leading to transcriptional changes in transporter genes that are responsible for Mn efflux.

To test this hypothesis, we analyzed the expressions of Mn transporter genes in transgenic Arabidopsis. The results showed that the overexpression of *GmMTP8.1* led to significant induction of *AtMTP11*, *AtNRAMP3*, and *AtECA3* in leaves of transgenic Arabidopsis under excess Mn conditions (Figure 9). In Arabidopsis, the Golgi-localized AtMTP11 and ECA3 are found to play crucial roles in Mn homeostasis by transporting Mn into the Golgi apparatus (Peiter et al., 2007; Mills et al., 2008), which might be further released to the extracellular space *via* the exocytosis of secretory vesicles, maintaining Mn homeostasis (Figure 10). Moreover, AtNRAMP3 is involved in the transportation of Mn from the vacuole into chloroplasts of mesophyll cells (Lanquar et al., 2010). Thus, the upregulation of these transporter genes has possibly contributed to stimulation of the export of Mn out of leaf cells in the *GmMTP8.1* overexpression lines. On the other hand, we found that Mn levels in roots of transgenic lines were similar to those in the WT under excess Mn conditions (Figure 8 and Supplementary Figure 7), and that the overexpression of *GmMTP8.1* led to increase in the transcript of *AtMTP8* in roots of transgenic Arabidopsis (Figure 9). The enhancement of *AtMTP8* may contribute to increase the sequestration of Mn into root vacuoles (Figure 10), although a direct measurement of intracellular Mn distribution would help to support this hypothesis.

In conclusion, a total of 14 *GmMTP* genes were identified in the soybean genome. These *GmMTPs* exhibited different responses to Mn toxicity. The heterologous expression of ER-localized *GmMTP8.1* facilitated the growth of yeast-sensitive mutant Δ*pmr1* and Arabidopsis under Mn toxicity conditions, which could be through the mechanisms of stimulating export of Mn out of cells and increasing sequestration of Mn in intracellular compartments.

## Data Availability Statement

The original contributions presented in the study are included in the article/Supplementary Material, further inquiries can be directed to the corresponding author/s.

## Author Contributions

ZC designed the research and revised the manuscript. JL, RD, YJ, JH, XZ, NA, and JS performed the experiments and analyzed the data. JL and RD wrote the manuscript. All the authors approved the final version of the manuscript.

## Conflict of Interest

The authors declare that the research was conducted in the absence of any commercial or financial relationships that could be construed as a potential conflict of interest.

## Publisher’s Note

All claims expressed in this article are solely those of the authors and do not necessarily represent those of their affiliated organizations, or those of the publisher, the editors and the reviewers. Any product that may be evaluated in this article, or claim that may be made by its manufacturer, is not guaranteed or endorsed by the publisher.

## Footnotes

1
http://blast.ncbi.nlm.nih.gov

2
https://phytozome-next.jgi.doe.gov/

3
http://www.expasy.org/tools.html

4
http://gsds.cbi.pku.edu.cn/

5
http://www.megasoftware.net/mega4/mega41.html

6
http://www.euroscarf.de/search.php?name=Order
