# Supplementary material for: Characterization of Metal Tolerance Proteins and Functional Analysis of GmMTP8.1 Involved in Manganese Tolerance in Soybean
Source: Front Plant Sci. 2021 Nov 29;12:683813. doi: 10.3389/fpls.2021.683813 (PMC8666509; doi:10.3389/fpls.2021.683813)
Supplement: Supplementary file 1 [file Data_Sheet_1.doc]

**Supplementary Table S1 Primers used for qRT-PCR and *GmMTP8.1* functional analyses.**

| **primer name** | **Forward primer (F, 5’-3’)** | **Reverse primer (R, 5’-3’)** |
| --- | --- | --- |
| *GmMTP4.1-RT* | CAAGGGAGTGCAGGATGTTC | ATGGAACTTATGCCAGGCTC |
| *GmMTP4.2-RT* | GCTGGAAAATGGCCTCCTAA | ATGGAACTTATGCCAGGCTC |
| *GmMTP8.1-RT* | GCGCATACACATTTGGGGTC | TGAACAAATGCCCGCTCAAC |
| *GmMTP8.2-RT* | CACTGTCCGTGCCTACACAT | TGAAGGCTCTCTCCAATGGC |
| *GmMTP8.3-RT* | TGACTTTATGGCTGGTGGCA | CTTGAAAGCCAAGTGTCGCC |
| *GmMTP8.4-RT* | CGTAGCTCGATCTCTTCTGTGT | TGAAACCATCCATGGAGTAAACA |
| *GmMTP8.5-RT* | ACAAGTGCAGTCTGAAAGGG | CAATGGGATACTCACCAGCC |
| *GmMTP9.1-RT* | GCTGCTATGCTTGCTGTCAA | TGGCGCTGTCCTTCCAATAA |
| *GmMTP10.1-RT* | ATCATCGTCAACATCACCGT | AGTTCCTGGTAAACCACACG |
| *GmMTP10.2-RT* | AACAGCACCGTTCCTTCACT | GGGAAACCACCCGTTTCAGT |
| *GmMTP10.3-RT* | GAGAGTGGCGGAGGAAATCG | GGCTTGGCAAACGAAACTCT |
| *GmMTP10.4-RT* | GGACCTCTTGTCAGGGTTCA | CACTGGTTGCATGCGTTTCT |
| *GmMTP11.1-RT* | CGGTGCTATCATTCTGGCTT | TATCAATGTGCCTCACAGCC |
| *GmMTP11.2-RT* | TTGATACGGTTCGGGCATAC | ACGCTCAATCTCAGGCAAAA |
| *GmMTP8.1-pYES2* | AGGGATCCAAAAAAATGTCTAAGAT  CTCTAATTATGC | GAATTCCTCAAGGCTGATTGTTGGG |
| *GmMTP8.1-GFP* | GAATTCATGAAGATCTCTAATTATGC | GGATCCTCAAGGCTGATTGTTG |
| *GmMTP8.1-OE* | GGATCCGATGGAAGGTGGTTTG | ACGCGTTTATATCTGGCTGCTGG |
| *GmEF1a-RT* | TGCAAAGGAGGCTGCTAACT | CAGCATCACCGTTCTTCAAA |
| *AtIRT1-RT* | AAATGTTCGAAGGCATGGGT | CGATCCCTAACGCTATTCCG |
| *AtMTP8-RT* | TACCGTCCGTGCATATACCT | GCTCTTTCCACCTCAGGAAG |
| *AtMTP11-RT* | GTTTGGGTCCGGAAGACAAT | TCGCCAATGTCTCGCTTTTA |
| *AtCAX2-RT* | AAGTTGAAATGGTGTCGGCT | TTCTTTGGTGCTTTAGGCGT |
| *AtCAX4-RT* | TCTCGGCACCAAATTGTTCA | GGACCGGTATGGAAAGCAAT |
| *AtCAX5-RT* | GTTGCCTATGCTGCTTACCT | GCATCAACAAGATAGCCGGA |
| *AtNramp1-RT* | CGTTCCGCTTCTGGTATCAA | GGCTTAAGTTGGGGGCATTA |
| *AtNramp3-RT* | AATATGGAGGCGGTGTGTTC | CCATTATGAACTGTCCCGCA |
| *AtNramp4-RT* | TGAGCAAATCATGGGCAGTT | CAAATTCGTTGCAGCTCCTG |
| *AtZIP1-RT* | CATGGTTCGACCGAGTTGAT | AGCCATGAGTGGCTTTATGG |
| *AtZIP2-RT* | CATCTTTGAGGGAATCGCCA | CCAAAGGCGAAGGAGTAGAC |
| *AtHMA2-RT* | AAGCATTCCCCGTGCCTAAA | CATCTTCGCAACCACGCAAT |
| *AtHMA4-RT* | TAACGGGCGAAGCATTTCCT | AGCCATTTTCGCAACCACAC |
| *AtYSL4-RT* | TCGGTCTCTTCTGTCTCGTT | GCAAGCTCAGCTCCAGTATT |
| *AtYSL6-RT* | ACTTGTGTTGTCGCTTGCTA | AGAGTCCCAAGAAGCTGACT |
| *AtECA1-RT* | GCAACGGTGGGAGTATTCAT | GAGAAAGTCTGAGAGCCTGC |
| *AtECA3-RT* | GCAGTGACAAGACTGGAACA | CCAGATTGTCAAAAGCCCCT |
| *AtEF1a-RT* | GTCGATTCTGGAAAGTCGACC | AATGTCAATGGTGATACCACGC |

**Supplementary Table S2** General information for the *GmMTP* genes in soybean.

| **Gene Name** | **Gene Locus** | **Chromosomal Location** | **CDS Length (bp)** | **AA** | **MW (kDa)** | **p*I*** | **TMD** | **Identity to ShMTP8 (%)** |
| --- | --- | --- | --- | --- | --- | --- | --- | --- |
| *GmMTP4.1* | Glyma.11G135500 | Chr11:10310228-10312318 | 1188 | 395 | 39.5 | 6.0 | 6 | 9.9 |
| *GmMTP4.2* | Glyma.12G059000 | Chr12:4270381-4272387 | 1167 | 388 | 42.7 | 5.9 | 6 | 11.9 |
| *GmMTP8.1* | Glyma.18G050400 | Chr18:4353324-4358090 | 885 | 294 | 33.1 | 6.5 | 4 | 90.8 |
| *GmMTP8.2* | Glyma.02G237600 | Chr02:42597212-42600960 | 1281 | 426 | 47.9 | 5.3 | 5 | 60.2 |
| *GmMTP8.3* | Glyma.14G206600 | Chr14:47200208-47203172 | 1179 | 392 | 47.1 | 6.7 | 3 | 67.9 |
| *GmMTP8.4* | Glyma.03G146700 | Chr03:36196590-36203201 | 1197 | 398 | 44.5 | 5.4 | 3 | 67.8 |
| *GmMTP8.5* | Glyma.19G150000 | Chr19:41018746-41025024 | 1074 | 357 | 40.3 | 5.4 | 3 | 62.7 |
| *GmMTP9.1* | Glyma.09G099500 | Chr09:18099034-18105167 | 1233 | 410 | 47.1 | 6.7 | 4 | 46.1 |
| *GmMTP10.1* | Glyma.08G164700 | Chr08:12972477-12975940 | 966 | 321 | 36.9 | 7.3 | 2 | 37.4 |
| *GmMTP10.2* | Glyma.15G262400 | Chr15:49495728-49499550 | 1194 | 397 | 45.6 | 7.9 | 5 | 47.1 |
| *GmMTP10.3* | Glyma.08G164800 | Chr08:12978532-12982936 | 1191 | 396 | 45.3 | 7.3 | 5 | 47.5 |
| *GmMTP10.4* | Glyma.15G262300 | Chr15:49484391-49488666 | 1191 | 396 | 45.6 | 8.3 | 5 | 47.7 |
| *GmMTP11.1* | Glyma.02G095400 | Chr02:8561703-8565611 | 1191 | 396 | 44.9 | 5.0 | 4 | 47.2 |
| *GmMTP11.2* | Glyma.18G286100 | Chr18:56628728-56632642 | 1191 | 396 | 44.9 | 5.1 | 4 | 46.7 |

Note, Gene locus, chromosomal location and CDS length of *GmMTPs* were obtained from Phytozome website. AA, Length of the amino acid sequence. MW, Molecular weight of the amino acid sequence. p*I*, Isoelectric point of the GmMTP proteins. TMD, Number of transmembrane domains. The homology identity (%) between each GmMTP protein and ShMTP8 from *Stylosanthes hamata* was determined by ClustalW (https://www.genome.jp/tools-bin/clustalw).

**Supplementary Table S3** Conserved motifs in GmMTP proteins.

| **Motif** | **Length** | **Protein sequences** | **Pfam Domain** |
| --- | --- | --- | --- |
| 1 | 61 | RAYTFGVHYFVEVDIVLPEDMPLKZAHNIGESLQEKLEQLPEVERAFVHJDFECTHKPEHS | ZT_dimer |
| 2 | 80 | NAANLVLFAAKVYASVMKNPNQYKYPIGRSGSJAVIASTLDSLLDLLSGFILWFTALAKKRMQPVGIIVFASVMATLGLQ | Cation_efflux |
