# Supplementary material for: Characterization of Metal Tolerance Proteins and Functional Analysis of GmMTP8.1 Involved in Manganese Tolerance in Soybean
Source: Front Plant Sci. 2021 Nov 29;12:683813. doi: 10.3389/fpls.2021.683813 (PMC8666509; doi:10.3389/fpls.2021.683813)
Supplement: Supplementary file 2 [file Data_Sheet_2.doc]

Supplementary Figure S1

*GmMTP10.1*

*GmMTP10.2*

*GmMTP10.3*

*GmMTP10.4*

*GmMTP9.1*

*GmMTP11.1*

*GmMTP11.2*

*GmMTP8.4*

*GmMTP8.5*

*GmMTP8.1*

*GmMTP8.2*

*GmMTP8.3*

*GmMTP4.1*

*GmMTP4.2*


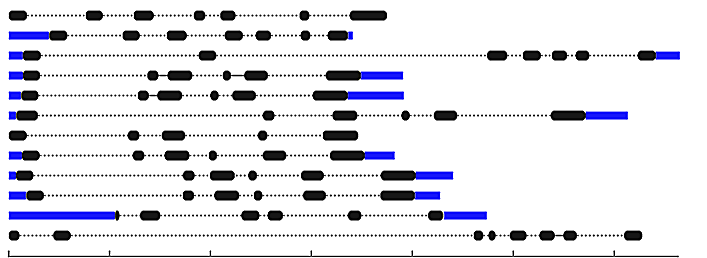


0 1 2 3 4 5 6 kb

5’

3’


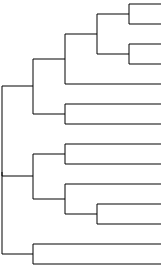

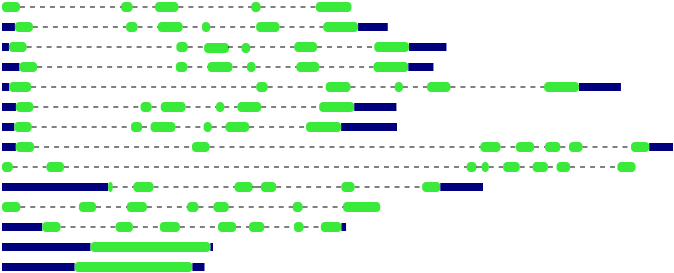


Exon

Upstream/downstream region

Intron


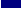


**Supplementary Figure S1** Neighbor-joining phylogenetic tree and gene structure of *GmMTPs*. The neighbor-joining phylogenetic tree of *GmMTP* gene sequences was constructed using MEGA 4.1. Gene structure of *GmMTPs* was generated from the Gene Structure Display Server. The light green and dark dotted lines indicate exons and introns, respectively. The blue boxes indicate the upstream/downstream regions of *GmMTPs*.

Supplementary Figure S2

Fe Zn Cu

*GmMTP4.1*

*GmMTP4.2*

*GmMTP8.1*

*GmMTP8.2*

*GmMTP8.3*

*GmMTP8.4*

*GmMTP8.5*

*GmMTP9.1*

*GmMTP10.1*

*GmMTP10.2*

*GmMTP10.3*

*GmMTP10.4*

*GmMTP11.1*

*GmMTP11.2*

*GmMTP4.1*

*GmMTP4.2*

*GmMTP8.1*

*GmMTP8.2*

*GmMTP8.3*

*GmMTP8.4*

*GmMTP8.5*

*GmMTP9.1*

*GmMTP10.1*

*GmMTP10.2*

*GmMTP10.3*

*GmMTP10.4*

*GmMTP11.1*

*GmMTP11.2*

B

Fe Zn Cu

15

10

5

0

-5

-10


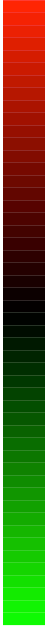


A

4

2

0

-2

-4

-6


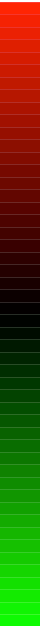

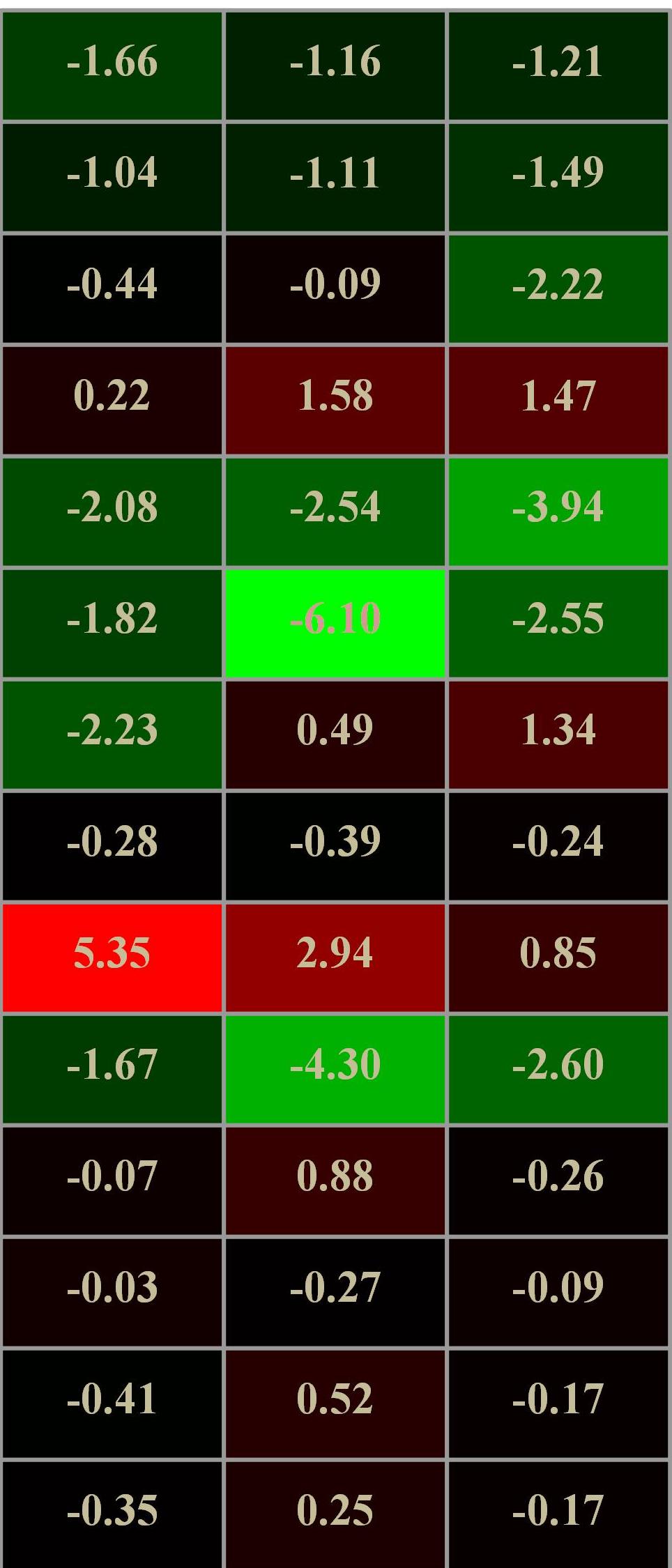

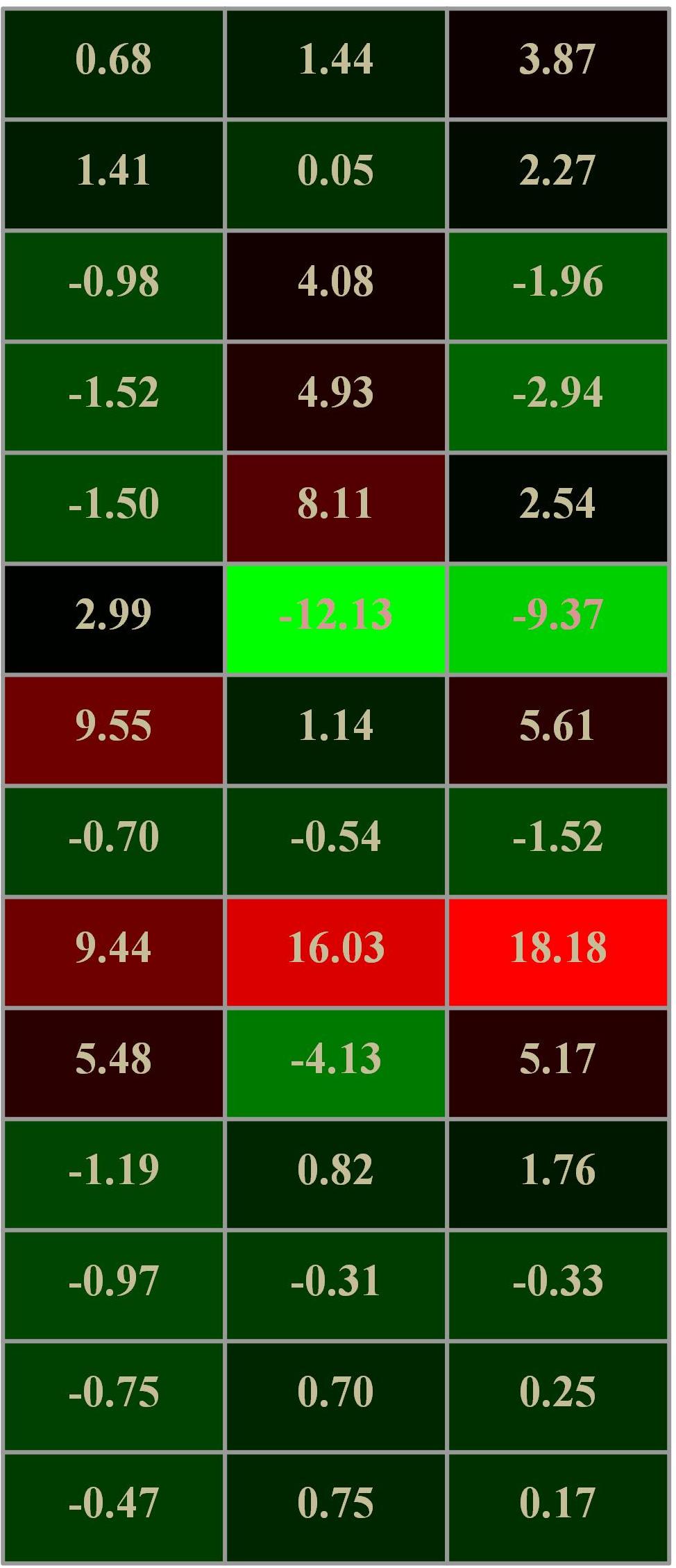


**Supplementary Figure S2** Expression analysis of *GmMTPs* in leaf **(A)** and root **(B)** of soybean at excess Fe, Zn and Cu treatments. Fourteen-day-old soybean seedlings were transplanted to 800 μM Fe, 20 μM Zn and 20 μM Cu treatments for 7 d. Data are expressed as the binary logarithm of fold-changes of relative expression of *GmMTP* members under metal stresses compared with their respective controls. The color scale bars ranging from red to green indicate high and low expression, respectively. The absolute value of data shown in the figure > 0.5 was regarded as significant difference.

Supplementary Figure S3

A


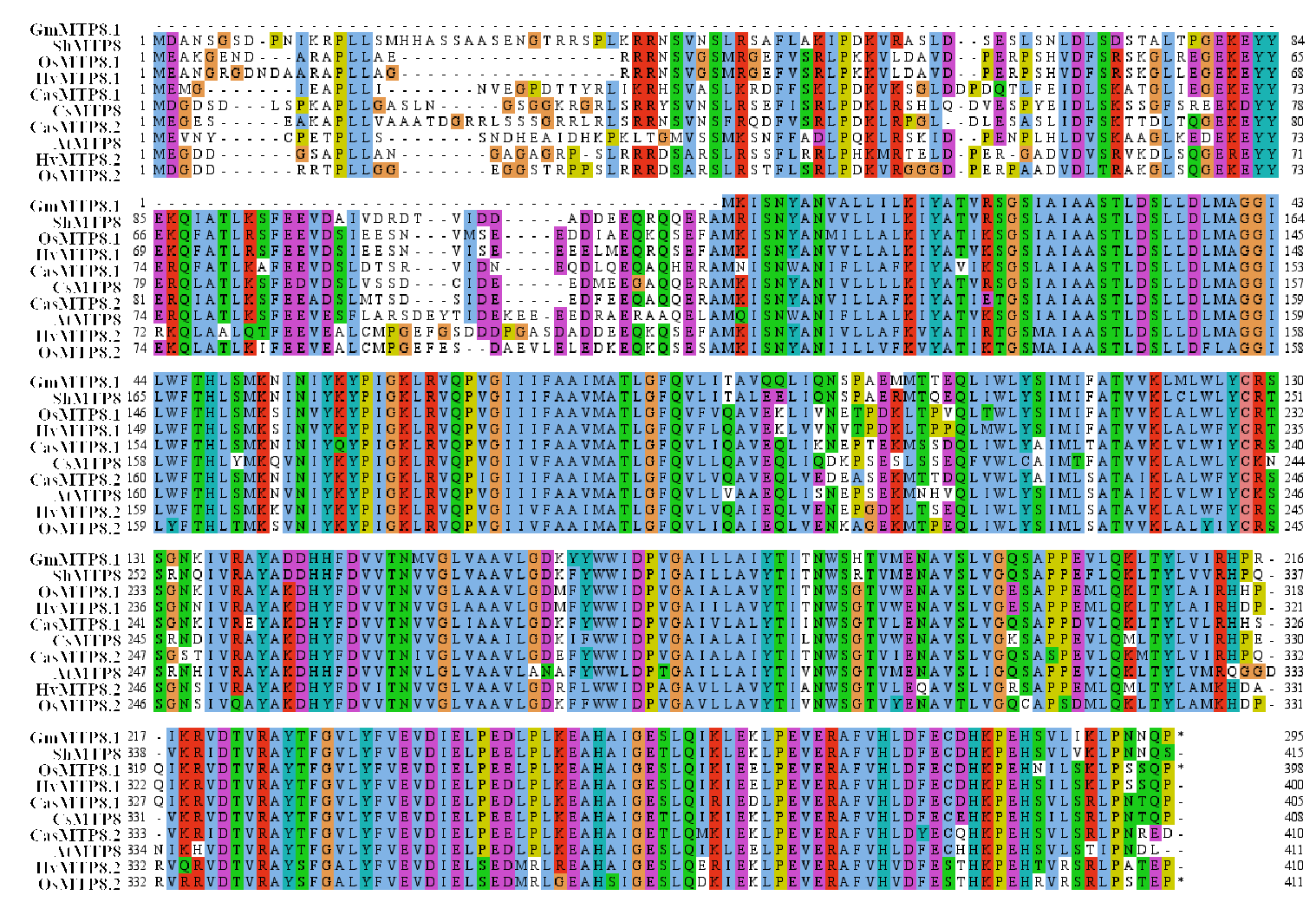


*********

*********

**I**

**II**

**III**

**IV**

B

**Supplementary Figure S3** Sequence alignment and homology identity of GmMTP8.1 with other MTP8-like proteins in plants. (**A**) Sequence alignment of GmMTP8.1 with those closely related MTP8-like proteins in plants. The transmembrane domains of GmMTP8.1 labeled with lines above the sequence were predicted by the HMMTOP2.0 program. The signature sequence of the Mn-CDF family is boxed. Asterisks below the sequence indicate the conserved sequence (DxxxD). Except for CasMTP8.1 and CasMTP8.2 derived from tea plant (*Camellia sinensis*), the first two letters of each protein represent the abbreviated species name. At, *Arabidopsis thaliana*; Gm, *Glycine max*; Sh, *Stylosanthes hamata*; Os, *Oryza sativa*; Cs, *Cucumis* *sativus*; Hv, *Hordeum vulgare*. (**B**) Homology identity of GmMTP8.1 with those closely related MTP8-like proteins in plants. The identity (%) was determined by ClustalW (https://www.genome.jp/tools-bin/clustalw).

1. AtMTP8

Supplementary Figure S4

A


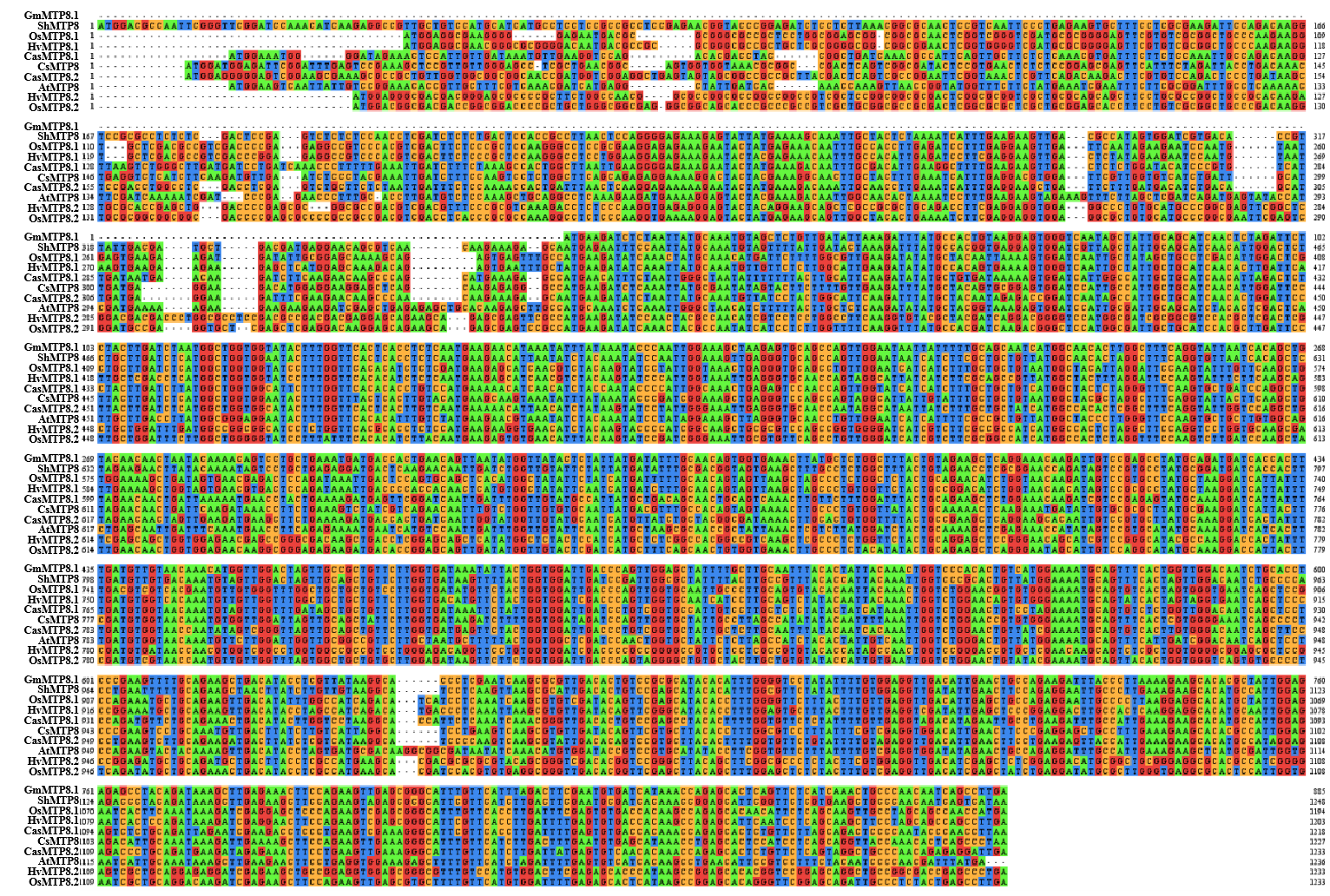


1. OsMTP8.2
2. HvMTP8.2

B

**Supplementary Figure S4** Sequence alignment and homology identity of *GmMTP8.1* with other *MTP8-like* genes in plants. (**A**) Sequence alignment of *GmMTP8.1* gene with those closely related *MTP8-like* genes. Except for *CasMTP8.1* and *CasMTP8.2* derived from tea plant (*Camellia sinensis*), the first two letters of each protein represent the abbreviated species name. At, *Arabidopsis thaliana*; Gm, *Glycine max*; Sh, *Stylosanthes hamata*; Os, *Oryza sativa*; Cs, *Cucumis* *sativus*; Hv, *Hordeum vulgare*. (**B**) Homology identity of *GmMTP8.1* with those closely related *MTP8-like* genes in plants. The identity (%) was determined by ClustalW (https://www.genome.jp/tools-bin/clustalw).

Supplementary Figure S5

B

A

C

**Supplementary Figure S5** Homology identity of GmMTP8.1 with Arabidopsis AtMTP and other soybean MTP8-like proteins. (**A**) Identity of GmMTP8.1 with AtMTP proteins. (**B**) Identity of *GmMTP8.1* with *AtMTP* genes. (**C**) Identity among soybean MTP8-like members. The first two letters of each protein represent the abbreviated species name. At, *Arabidopsis thaliana*; Gm, *Glycine max*. The identity (%) was determined by Clustalw (https://www.genome.jp/tools-bin/clustalw).

Supplementary Figure S6

A

0.3

0.2

0.1

0

***

*

***

*

*GmMTP8.1*

Relative expression

WT OE1 OE2

B

1. WT OE1 OE2

(KDa)


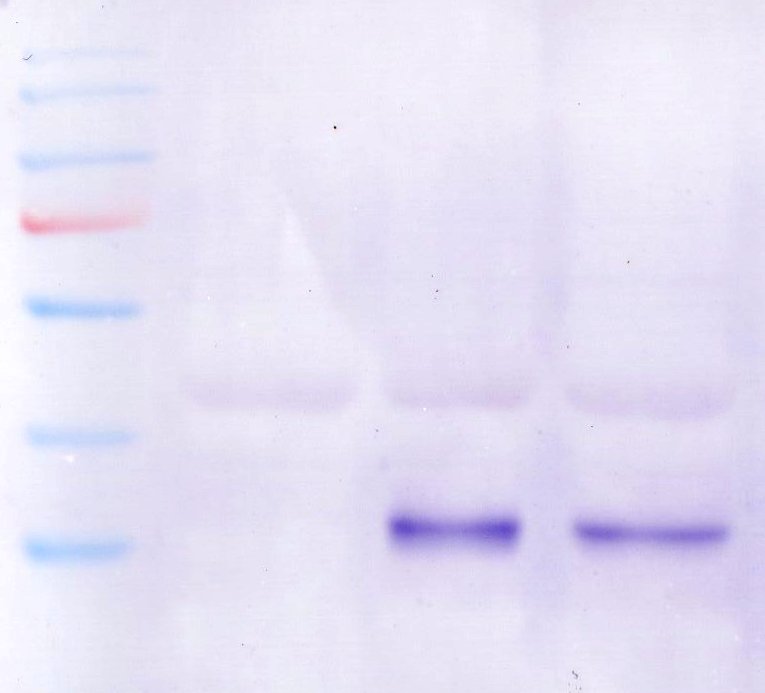

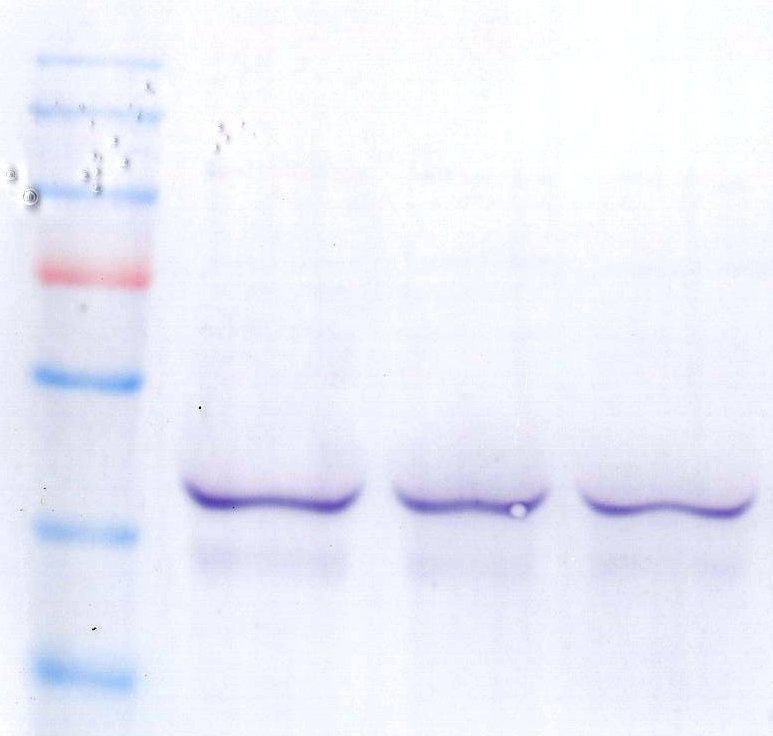


100

75

60

45

Anti-HPT

35

Anti-UGPase

45

**Supplementary Figure S6** Quantitative real-time (qRT) PCR **(A)** and Western blot **(B)** analyses of transgenic Arabidopsis overexpressing *GmMTP8.1*. Protein level of GmMTP8.1 indicated by hygromycin phosphotransferase was detected using an anti-hygromycin phosphotransferase (HPT) antibody. The anti-UGPase antibody was used as a control. Fourteen-day-old wild type and transgenic Arabidopsis were used. WT represents the wild type Arabidopsis. OE1 and OE2 are two independent transgenic lines overexpressing *GmMTP8.1*. Asterisks indicate significant differences between the wild type and the overexpression lines. ***, *P*<0.001.

Supplementary Figure S7

Shoot fresh weight

(g plant^-1^)

WT

OE1

OE2

Mn concentrations (µM)

Root fresh weight

(g plant^-1^)

18 400

Mn concentrations (µM)

18 400

Mn concentrations (µM)

18 400

Mn concentrations (µM)

18 400

Mn concentration in shoot

(mg g^-1^ DW)

Mn concentration in root

(mg g^-1^ DW)

B

C

D

E

400 µM [MnSO_4_]

18 µM

WT OE1 OE2


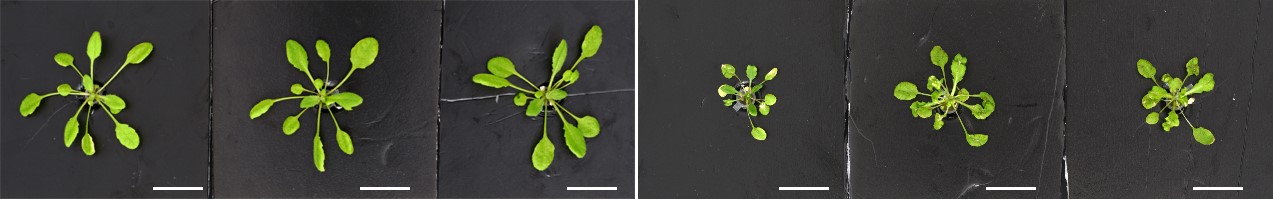


WT OE1 OE2

A

*

*

**

**

**Supplementary Figure S7** Effects of excess Mn on the growth of transgenic Arabidopsis under hydroponic condition. **(A)** Phenotype of Arabidopsis grown under 18 and 400 µM Mn. **(B)** Shoot fresh weight. **(C)** Root fresh weight. **(D)** Mn concentration in shoot. **(E)** Mn concentration in root. 21-day-old Arabidopsis seedlings were transplanted into hydroponic nutrient solution containing 18 and 400 µM MnSO_4_ as control and excess Mn treatments, respectively. After 7 d of Mn treatments, fresh weight and Mn concentration were determined. WT represents the wild type Arabidopsis. OE1 and OE2 are two transgenic lines overexpressing *GmMTP8.1*. Each bar represents mean value of three independent replicates with standard error. White, gray and black bars represent WT, OE1 and OE2, respectively. Asterisks indicate significant differences between the wild type and the overexpression lines at the same Mn treatment. *, 0.01<*P*<0.05. **, 0.001<*P*<0.01. DW, Dry weight. Scale bar is 2 cm.
